# AFLP-AFLP *in silico*-NGS approach reveals polymorphisms in repetitive elements in the malignant genome

**DOI:** 10.1371/journal.pone.0206620

**Published:** 2018-11-08

**Authors:** Jitka Koblihova, Klara Srutova, Monika Krutska, Hana Klamova, Katerina Machova Polakova

**Affiliations:** Institute of Hematology and Blood Transfusion, Prague, Czech Republic; University of Helsinki, FINLAND

## Abstract

The increasing interest in exploring the human genome and identifying genetic risk factors contributing to the susceptibility to and outcome of diseases has supported the rapid development of genome-wide techniques. However, the large amount of obtained data requires extensive bioinformatics analysis. In this work, we established an approach combining amplified fragment length polymorphism (AFLP), AFLP *in silico* and next generation sequencing (NGS) methods to map the malignant genome of patients with chronic myeloid leukemia. We compared the unique DNA fingerprints of patients generated by the AFLP technique approach with those of healthy donors to identify AFLP markers associated with the disease and/or the response to treatment with imatinib, a tyrosine kinase inhibitor. Among the statistically significant AFLP markers selected for NGS analysis and virtual fingerprinting, we identified the sequences of three fragments in the region of DNA repeat element OldhAT1, LINE L1M7, LTR MER90, and satellite ALR/Alpha among repetitive elements, which may indicate a role of these non-coding repetitive sequences in hematological malignancy. SNPs leading to the presence/absence of these fragments were confirmed by Sanger sequencing. When evaluating the results of AFLP analysis for some fragments, we faced the frequently discussed size homoplasy, resulting in co-migration of non-identical AFLP fragments that may originate from an insertion/deletion, SNP, somatic mutation anywhere in the genome, or combination thereof. The AFLP–AFLP *in silico*–NGS procedure represents a smart alternative to microarrays and relatively expensive and bioinformatically challenging whole-genome sequencing to detect the association of variable regions of the human genome with diseases.

## Introduction

Efforts to explore the human genome through individual whole-genome analyses and identify genetic risk factors contributing to the susceptibility to and outcome of diseases are of vital importance. Since the costs of sequencing have become more reasonable, genome-wide techniques have evolved rapidly. However, the broad range of sequencing technologies incurs limitations due to the large volume of obtained data, requiring extensive bioinformatics analysis that involves working with a variety of genomic tools and databases [1,2]. Thus, it is sometimes difficult to mine key information and achieve an accurate interpretation of the resulting data.

A draft human genome sequence providing the first overview of the genome was published in 2001 [3] by the International Human Genome Sequencing Consortium, followed by completion and final publication 3 years later [4]. The pilot phase of the 1000 Genomes Project [5] was conducted to deeply characterize sequence variations, including the location, allele frequency and local haplotype structure of genetic variants, using a combination of low-coverage whole-genome sequencing, high-coverage sequencing, and exon-targeted sequencing of individuals from different populations. In this project, more than 95% of common variants (> 5% frequency), including 15 million SNPs, were identified. Low-coverage whole-genome (less than 6× coverage) and exome sequencing of 1,092 healthy individuals from 14 populations helped to increase knowledge of the genetic contribution to diseases [6]. A combination of current methods such as single-molecule real-time DNA sequencing (SMRT) was used to close or extend 55% of the remaining interstitial gaps in the human genome, including repetitive (short and long tandem repeats) and GC-rich regions in the human GRCh37 reference genome, and to characterize many structural variations, such as insertions/deletions, duplications and inversions [7]. Additionally, chromatin immunoprecipitation followed by sequencing analysis (ChIP-seq) is a promising tool for analyzing many samples simultaneously, although, no optimal single workflow for all samples exists, and large amounts of starting material (~10^5^ cells) and good quality management are required [8].

The application of high-throughput sequencing approaches to malignant genomes is at the forefront of genome research. The evaluation of data obtained through multiplatform analyses (whole-genome sequencing, whole-exome DNA sequencing, RNA and microRNA sequencing, and analyses of DNA copy-number variation and DNA methylation) of thousands of cancers belonging to 12 tumor types from The Cancer Genome Atlas project, including hematological malignancy–acute myeloid leukemia genomes [9], resulted in the classification of cancer into 11 major subtypes and, together with tissue-of-origin information, enhanced the prediction of clinical outcomes [10].

In this work, we established an approach combining amplified fragment length polymorphism (AFLP) and next-generation sequencing (NGS) techniques to map the malignant genomes of patients with chronic myeloid leukemia (CML), a disease characterized by the presence of reciprocal translocation between chromosomes 9 and 22 t(9;22)(q34;q11), known as the Philadelphia chromosome, resulting in a BCR-ABL1 fusion oncogene. It has been suggested that the first hit leading to CML development is a mutagenic event leading to the transformation of a normal hematopoietic stem cell to a premalignant progenitor cell population and subsequent generation of the BCR-ABL1 oncogene [11]. However, the mechanism underlying the formation of the Philadelphia chromosome and clonal expansion of pre-CML progenitor cells is still unknown [12–14]. The development of tyrosine kinase inhibitors (TKIs), such as imatinib, inhibiting the activity of the BCR-ABL1 tyrosine kinase, represented a significant breakthrough in CML treatment. The overall survival of patients treated with imatinib longer than 5 years is between 83% and 97%; however, 20–30% of patients are resistant to this therapy, resulting in therapeutic failure [15]. Fortunately, second- and third-generation TKIs, such as nilotinib, dasatinib, bosutinib, and ponatinib, have been successfully introduced in the treatment of CML and have been efficient in overcoming imatinib failure. One of the best-studied mechanisms of TKI resistance is the development of point mutations in the kinase domain of the BCR-ABL1 fusion gene. Other mechanisms of resistance, such as amplification of the BCR-ABL1 gene [13], loss of kinase target dependence [14], and pharmacogenomics, are still a subject of interest and remain to be elucidated.

The AFLP technique (Figure A in S1 File) is based on the selective PCR amplification of fragments from digested genomic DNA, as described by Vos et al. [16], and the whole procedure generates unique DNA fingerprints for each individual. Genetic differences among individuals lead to different sizes and numbers of fragments. Each DNA fragment in a profile is referred to as a locus or marker and is characterized by its size in base pairs. Since AFLP generates approximately 100 DNA fragments per individual, it is not feasible to isolate a single DNA fragment of interest from a gel according only to length. In the past, the DNA sequence was obtained after time-consuming cloning of fragments, whereas in the present study, we used NGS, which enables the identification and characterization of a particular AFLP marker in a sample containing several fragments of similar lengths.

The main advantages of using AFLP are that there is no requirement for prior knowledge of the genome sequence, and it makes use of randomly distributed markers throughout the genome. Moreover, this method combines the high reproducibility of DNA fingerprints (Figures B and C in S1 File) [17] and robustness with a relatively low-cost analysis. Differences in AFLP patterns and the presence/absence of AFLP markers mainly provide useful information about genetic diversity in analyses of plant genetics, although this methodology also plays an irreplaceable role in other fields, such as microbiology and virology [18–21]. Zhang et al. [22] reviewed advances in AFLP-based studies in combination with NGS for analysis of non-model genomes. However, very little data from the application of AFLP to the human genome has been published to date [23].

In this work, we described the AFLP fingerprinting of a cohort of patients and healthy donors, which enabled the identification of DNA regions associated with traits such as the disease itself and its response to the treatment, and we preselected loci in the human genome for further identification through next-generation sequencing. No extensive bioinformatic evaluation of the data was necessary to reveal the sequence of interest, in contrast to whole-genome or whole-exome sequencing. Thus, based on our results, we suggest that simple DNA fragmentation in combination with NGS is an advantageous and feasible approach for identifying genomic loci associated with human diseases.

## Materials and methods

### Patient characteristics

The study was approved by the Ethics Committee of the Institute of Hematology and Blood Transfusion in Prague. Written informed consent was obtained from all patients, in accordance with the Declaration of Helsinki.

We analyzed the genome of 65 patients at the time of diagnosis in the chronic phase of chronic myeloid leukemia (CP-CML), who were treated at the Institute of Hematology and Blood Transfusion in Prague, as well as 30 healthy donors. Another 30 healthy donors were added for Sanger sequencing analysis to confirm the AFLP-CML markers. All individuals were Europeans. The median age of the patients was 55 years (range 18–84 years), including 25 women (38%) and 40 men (62%). According to the recommendations of the European LeukemiaNet (ELN), the patients were divided into two groups based on their response to imatinib treatment at 12 months after therapy initiation [15]. Patients with optimal responses to imatinib therapy (N = 39) achieved a major molecular response after 12 months (MMR; BCR-ABL1^IS^ ≤ 0.1%; IS = International Scale). The second group consisted of patients exhibiting treatment failure, or patients in the “Warning” category (N = 26). At this time, treatment failure was not associated with the development of resistance to imatinib due to mutation in the kinase domain of BCR-ABL1, except in one patient in whom a T315I mutation was detected through Sanger sequencing at 9 months after therapy initiation (4 patients developed mutations after 12 months of imatinib treatment). All 65 patients were treated with the standard daily dose of imatinib (400 mg/day) for 12 months after therapy initiation. Patient characteristics, including clinical and molecular genetic parameters, are summarized in Table A in S1 File.

### AFLP analyses

Total genomic DNA was isolated from leukocyte guanidine isothiocyanate (SERVA Electrophoresis GmbH, Heidelberg, Germany) or TRIzol (Thermo Fisher Scientific, Waltham, MA, USA) lysates of peripheral blood cells deposited in the bio-bank. AFLP analyses were performed according to the manufacturer’s “AFLP Plant Mapping” protocol (Thermo Fisher Scientific; http://www3.appliedbiosystems.com/cms/groups/mcb_support/documents/generaldocuments/cms_040959.pdf) and the whole procedure is illustrated in Figure A in S1 File. All enzymes were obtained from Thermo Fisher Scientific, except for restriction endonucleases, which were purchased from New England Biolabs (Ipswich, MA, USA). Briefly, 200 ng of genomic DNA was digested using the restriction endonucleases EcoRI (5 U) and MseI (1 U), followed by adaptor ligation at 37°C for 2 h (AFLP Ligation and Preselective Amplification Kit for Regular Plant Genomes). The products were then diluted 1:2 with TE_0.1_ buffer (20 mM Tris-HCl, 0.1 mM EDTA, pH 8.0) and used for preselective amplification. The products of preselective amplification were diluted 1:5 with TE_0.1_ buffer and served as templates for selective PCR using combinations of 8 MseI primers and 8 fluorescent dye-labeled (FAM, NED, JOE) EcoRI primers, as shown in Table 1. Suitable combinations of primers were selected based on the quality of the signal and the number of observed fragments after separation on an ABI PRISM 3130 Genetic Analyzer (Figure D in S1 File). Selective amplification was performed in a total volume of 10 μl (1.5 μl diluted preselective amplification reaction product, 0.5 μl MseI at 5 μM, 0.5 μl EcoRI at 1 μM, 7.5 μl AFLP Core Mix). The quality of the restriction digests, ligation of adaptors, and products of preselective amplification for all samples was assessed by electrophoresis in a 2% agarose gel. The final PCR products were separated on an ABI PRISM 3130 Genetic Analyzer using GeneScan 500 ROX dye as a size standard (Thermo Fisher Scientific). The resulting DNA fingerprints were analyzed with GeneMapper software v4.1 (Thermo Fisher Scientific) and transformed to binary data 0/1, representing the absence/presence of a DNA fragment.

**Table 1:** Combinations of MseI (CXX) and fluorescent dye-labeled EcoRI (AXX) primers used for AFLP analyses.

EcoRI AXX (fluorescent dye)	MseI CXX							
	CTG	CTA	CTT	CAG	CAC	CAT	CTC	CAA
ACA (FAM)	x	x	x	x	x	x	x	x
ACT (FAM)	x	x	-	x	x	x	x	x
ACG (JOE)	x	x	-	x	-	-	x	x
AAG (JOE)	-	-	x	-	x	x	x	x
AGG (JOE)	x	x	-	x	x	-	x	x
ACC (NED)	x	x	x	x	x	x	x	x
AGC (NED)	x	x	-	-	-	-	x	x
AAC (NED)	x	x	x	x	x	x	x	x

x—used combination of primers,—primer combinations unsuitable for analysis due to a low signal or small number of generated fragments

### Statistical analyses

Binary data from the AFLP analyses were statistically evaluated using cluster analysis via the unweighted neighbor-joining method in DARwin software v6.0 [24], to construct hierarchical trees. The statistical significance of AFLP markers associated with CML or the response to imatinib treatment was assessed via Fisher´s exact probability test, using on-line utilities available at http://vassarstats.net, after fragment analysis (predicted statistics) and Sanger sequencing (real statistics). P values < 0.05 indicate a significant difference.

### AFLP *in silico*

The AFLP markers that were statistically significant for patients with CP-CML or the treatment response and were selected for further analysis through NGS were also analyzed *in silico* to predict AFLP fragments of corresponding lengths that might be generated during the standard AFLP procedure. We used freely available software (http://www-leca.ujf-grenoble.fr/moyens-techniques/logiciels/article/population-genomics-software?lang=fr) to perform *in silico* fingerprinting (ISIF) (Paris et al. 2010). DNA digestion was simulated using the MseI and EcoRI restriction endonucleases for the human genome sequences of all chromosomes, downloaded from ftp://ftp.ncbi.nih.gov/genomes/Homo_sapiens. The obtained sequences were then mapped to chromosomes using public databases and screened for indels or SNPs responsible for the introduction/loss of MseI or EcoRI restriction sites. If no indel/SNP was found in the predicted sequence, we simulated the presence of a SNP/indel in both restriction sites and the selective bases of the MseI and EcoRI primers (48 substitutions/1 fragment). Using this approach, we did not simulate the presence of SNPs/indels leading to the introduction of a new restriction site inside the sequence between two selective bases of EcoRI and MseI primers, or the presence of more than one SNP or indel in the restriction sites or selective bases of the primers simultaneously.

### Identification of AFLP markers using NGS

Selective amplification reactions for selected AFLP fragments (N = 9) in representative samples were prepared in 8 x 10 μl, as described above, then pooled and concentrated to 6 μl using a SpeedVac SPD 111V P1 (Thermo Fisher Scientific). Next, the samples were subjected to electrophoresis in a 3% agarose gel for 7 h at 50 V. Three gel regions were tested for each fragment (Figure E in S1 File). The region containing the greatest amount of the particular fragment and the smallest number of other undesired fragments was used for further processing. Gel regions containing DNA fragments of certain lengths ± 50 bp (according to the GeneRuler 50 bp DNA Ladder, Thermo Fisher Scientific) were excised and purified using the QIAEX II Gel Extraction Kit (Qiagen, Hilden, Germany). To assess the quality of isolation and confirm the presence of fragments of the required length, fragmentation was performed on an ABI PRISM 3130 Genetic Analyzer.

NGS was carried out on a GS Junior system (454 Sequencing Systems; 454 Life Sciences, Roche Applied Science, Branford, CT, USA). Preparation of the amplicon library was performed according to the manufacturer’s recommendations, with modifications in several steps. To enable the ligation of adaptors with molecular identifier tags (MIDs), the ends of purified DNA fragments were blunted using the GS FLX Titanium Rapid Library Preparation Kit (Roche Applied Science). Adaptors were added at a 100x lower concentration than indicated in the protocol to prevent self-ligation and further amplification during emulsion PCR. The samples were purified using Agencourt AMPure XP beads (Beckman Coulter, Brea, CA, USA) at a ratio of 1.8:1 (beads:sample). The presence/absence of self-ligated adaptors and amplified products was assessed using an Agilent High Sensitivity DNA Assay on an Agilent 2100 Bioanalyzer (Figure F in S1 File), with quantification via RT-qPCR (KAPA Library Quantification Kits; KAPABIOSYSTEMS). The products were then diluted and pooled in an equimolar ratio. Emulsion PCR was performed using the GS Junior Titanium emPCR Kit (Lib-L). Sequencing was performed according to the manufacturer’s protocol (Roche Applied Science).

The data from NGS were evaluated using NextGENe v2.4.1 software (Softgenetics, State College, PA, USA), and the obtained reads were aligned to the sequences of fragments predicted through AFLP *in silico* and used as reference sequences. Two approaches in NextGENe software were applied, the amplicon and shotgun sequencing. The number of reads provided by both approaches are listed in Table C in S1 File. Fragments of interest were recognized based on their lengths (determined from electrophoregrams), the presence of EcoRI (G|AATTC) and MseI (T|TAA) restriction sites and appropriate selective primer sequences (AXX for EcoRI primer; CXX for MseI primer; X stands for A, C, G or T) at the respective end of the sequence. The aligned sequences were then evaluated using open-source databases: Ensembl release 80—May 2015; NCBI; UCSC Genome Browser [25]; Dfam 1.4 (May 2015, 1306 entries) [26]; RepeatMasker 4.0.5 [27]. Minor allele frequencies (MAF) of SNPs originated from sequencing projects 1000 Genomes Project (1000 Genomes) [28], TOPMED or the Exome Aggregation Consortium (ExAC) [29].

### Sanger sequencing

To confirm the fragments identified through NGS, Sanger sequencing of 9 AFLP markers was performed. The sequences of all primers, designed to produce amplicons 413–940 bp in length, and their annealing temperatures are shown in Table E in S1 File. The primers were custom synthesized by Integrated DNA Technologies (Coralville, IA, USA). PCR was carried out using AccuPrime SuperMix I (Thermo Fisher Scientific) under the following conditions: 94°C 2 min (94°C 15 s, annealing temperature (°C–see Table E in S1 File) 30 s, 68°C 1 min) x 35 cycles. The products were purified with the QIAquick PCR Purification Kit (Qiagen), followed by sequencing PCR with the BigDye Terminator kit v.3.1 (Thermo Fisher Scientific): (96°C 10 s, 55°C 10 s, 60°C 4 min) x 25 cycles. The products were then purified with a DyeEx 2.0 Spin kit (Qiagen), dried using a SpeedVac SPD 111V P1 (Thermo Fisher Scientific), dissolved in 25 μl of formamide and denatured for 4 min at 96°C. Sequencing was performed on an ABI 3500 or ABI 3500xl Genetic Analyzer. Mutation Surveyor v3.10 software (Softgenetics, State College, PA, USA) was used for analysis of the obtained sequences.

### Linkage disequilibrium analyses

The linkage disequilibrium of the genotyped SNPs was assessed using the LDlink 1.1 database [30]. To generate an interactive heatmap matrix of pairwise linkage disequilibrium statistics, the alleles of a pair of SNPs in high LD were investigated, and proxy and putatively functional SNPs for a query SNP were explored. A European population group was chosen.

## Results

**Fig 1:**
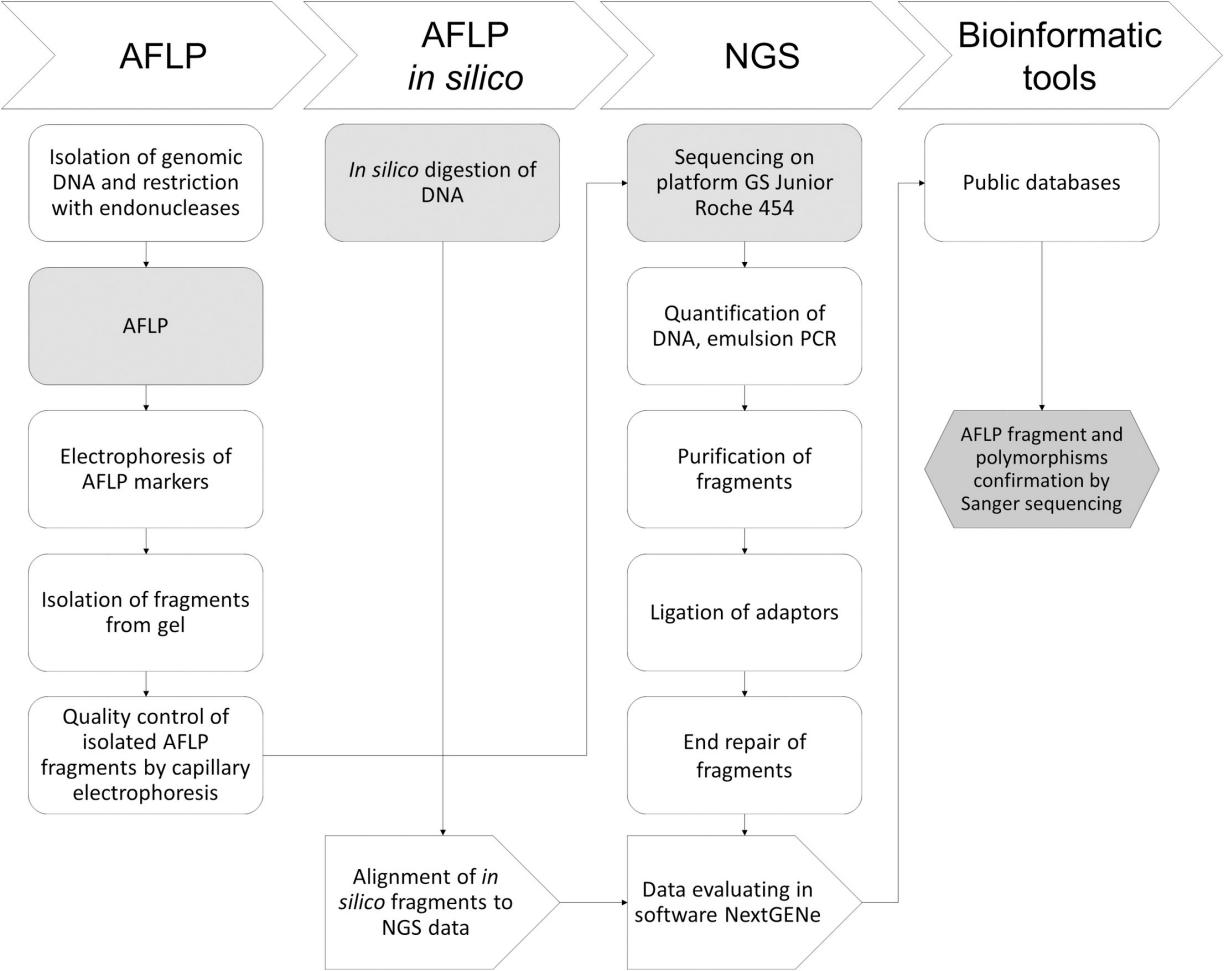
Workflow of newly established AFLP/NGS procedure for AFLP markers identification.

In this work, we established an approach combining AFLP, AFLP *in silico* and NGS methods to map the malignant genome of patients with chronic myeloid leukemia. The workflow of the AFLP-*AFLP in silico*-NGS approach is shown in Fig 1 and the particular results are mentioned further in the text.

### AFLP fingerprints clearly distinguished patients with CML from healthy individuals

We performed AFLP analyses of samples of 65 CML patients and 30 healthy donors using 51 primer combinations (Table 1). All 51 primer combinations, applied on all 95 samples analyzed, generated 4845 DNA individual profiles, i.e. fingerprints. Selective amplification with primer combinations generated 31 (CAG_ACG) to 112 (CAA_AGG, CTT_AAG, CTG_AAC, CAG_AAC) fragments that were separated according to their length by capillary electrophoresis. Overall, we detected and evaluated 3912 AFLP fragments or so-called AFLP markers per each individual patient profile.

**Fig 2:**
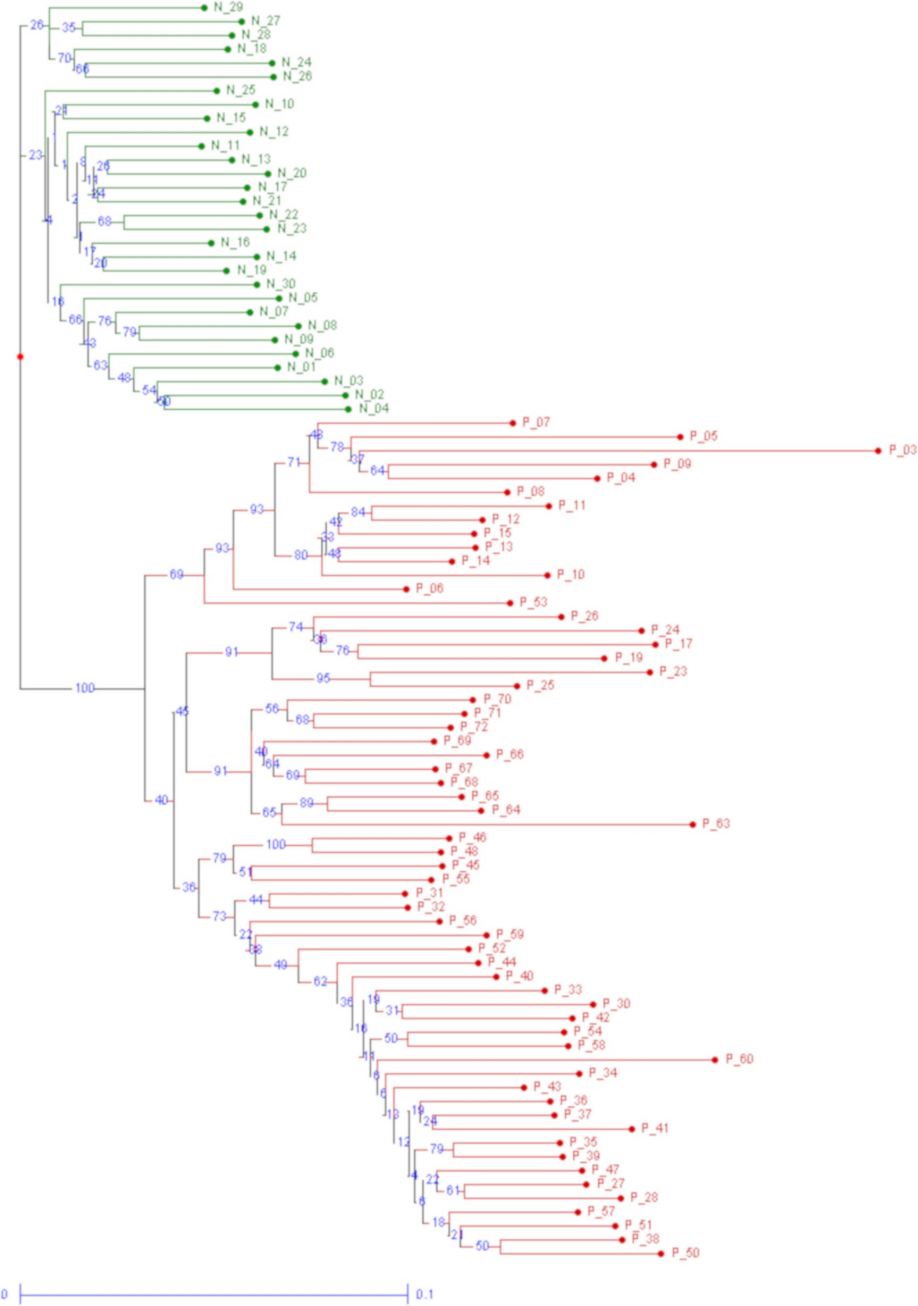
Hierarchical cluster analysis of all healthy controls and CML patients. Dendrogram illustrating two distant clusters of healthy donors (N = 30; green) and patients with CML (N = 65; red) was constructed based on the distance matrix calculated using the unweighted neighbor-joining method in DARwin software v6.0 [24]. The calculation was done with all 3912 AFLP markers detected after fragmentation analysis in all samples of healthy donors and CML patients.

In CML patients, no association was found between the AFLP markers and neither clinical nor molecular genetic parameters (e.g., sex, smoking status, Hasford risk, age at diagnosis, BCR-ABL1 oncogene at mRNA levels; Table A in S1 File), probably due to the low number of patients. However, Fisher´s exact probability test, comparing the presence/absence of AFLP markers between CML patients and healthy donors, enabled the selection of a set of AFLP markers detected significantly frequently in CML patients compared to healthy controls (N = 180; Table B in S1 File). Similarly were identified AFLP markers significantly frequently linked to CML patients resistant to imatinib treatment (N = 2; Table 2). Hierarchical cluster analysis of all AFLP fragments analyzed in CML patients and healthy donors, all European population, resulted in formation of 2 major distant clusters; one of healthy donors and the second of CML patients (Fig 2). This analysis highlighted that genome of leukemic cells differ from the normal leukocytes.

**Table 2:** AFLP markers selected for NGS analysis.

AFLP marker	Number of detected (1) or undected (0) fragments in all individuals analyzed	Probability that the fragment will be present or absent in the specific cohort of individuals				Intensity of signal	Fragment length (bp)
**Sex**	** **	**CML women**	**P =**	**CML men**	**P =**	** **	** **
CAG_ACC_32	1 (N = 38)	0	2.59E-10	38	4.73E-08	++	188
	0 (N = 22)	22		0			
**Response to IM**	** **	**Patients sensitive to IM**	**P =**	**Patients resistant to IM**	**P =**	** **	** **
CTT_ACA_57	1 (N = 29)	24	0.0306	5	0.0056	++	257
	0 (N = 34)	13		21			
CAG_AGG_56	1 (N = 30)	23	0.1017	7	0.0460	+++	263
	0 (N = 32)	14		18			
**CML**	** **	**Controls**	**P =**	**Patients**	**P =**	** **	** **
CAA_ACC_41	1 (N = 47)	29	6.03E-08	18	0.0006	+++	172
	0 (N = 46)	1		45			
CAC_ACC_25	1 (N = 42)	4	0.0003	38	0.0163	+++	152
	0 (N = 51)	26		25			
CAC_AAG_063	1 (N = 72)	29	0.0020	43	0.0619	+++	195
	0 (N = 19)	0		19			
CAA_AAC_50	1 (N = 75)	30	2.21E-03	45	0.0777	+	242
	0 (N = 18)	0		18			
CAA_ACC_19	1 (N = 23)	1	4.61E-03	22	0.0782	+++	104
	0 (N = 70)	29		41			
CAT_ACC_54	1 (N = 24)	0	0.0002	24	0.0431	++	174
	0 (N = 68)	29		39			
CAT_ACC_55*	1 (n = 54)	12	0.0618	42	0.2037	++	175
	0 (n = 38)	17		21			

Total number of patients in category “Response to IM” consists of data obtained from patients sensitive or resistant to imatinib. In category CML, samples of healthy donors and CML patients were examined. If the fragment analysis provided poor-quality spectra, data were excluded. An intensity of the signal was evaluated as +++ high, ++ medium, and + low.

* indicates the fragment that was not primarily sequenced by NGS, however, its sequence was determined using Sanger sequencing.

As one can expect, we should observe the AFLP fragment definitely distinguishing CML patients from healthy donors that would originated from the BCR-ABL1 fusion gene. It is known that BCR-ABL1 fusion gene is caused by reciprocal translocation of chromosomes 9 and 22. The breaks occur in intron 13 or 14 of BCR gene and intron 1 of ABL1 gene in majority of CML patients. Since the position of the break is patient-specific, it was unlikely that we could see statistically significant AFLP fragment of the same size in DNA fingerprints of several patients. Moreover, this assumption is supported by the *in silico* digestion (http://www.restrictionmapper.org/) of the intron sequences. We found that the restriction site G|AATTC for EcoRI restriction endonuclease does not occur either in intron 13 or intron 14 of BCR gene, thus the fragment would not be generated. We also definitely confirmed that by analyses of 19 patient (same as used for AFLP analyses) sequences, where we exactly know the break position and no restriction site for EcoRI was observed. The restriction site for EcoRI endonucleases could potentially introduce only with SNP(s).

### Sequence identification of selected AFLP markers through NGS

The statistical significance, quality and intensity of the signals observed in spectra after fragment analysis, together with the optimal fragment length of 100–300 bp, were the main criteria for the selection of fragments for further NGS analysis. The frequency of the presence of fragments and statistical significance (Fisher´s exact probability test), classified according to the response to imatinib therapy or CML diagnosis vs. healthy status for 9 selected AFLP markers, are shown in Table 2. We selected one dominant AFLP marker (i.e., CAG_ACC_32) and 8 co-dominant markers. The sequences obtained by NGS for which the results were in accordance with the results of the fragment analysis in terms of fragment length (bp), the estimated proportion of the AFLP marker in a sample expressed based on the number of reads, and their mapping to the reference sequence of the human genome are listed in Table 3. As a control for the whole analytical procedure, including the characterization and localization of AFLP markers, we added an AFLP marker associated with the Y sex chromosome, CAG_ACC_32, identified among 60 CML patients (Table 2) and 30 healthy donors. The 188-bp fragment was absent in women and present in men. Its sequence, characterized through NGS, was mapped to chromosome Y at 3 different positions and was part of the long terminal repeats (LTR) MER11B and MER11C (family ERVK). We also confirmed the sex of patients and healthy donors based on the presence of the CAA_ACC_12 fragment (92 bp) in the fragment analysis spectra that were in accordance with the results for CAG_ACC_32. We obtained the sequence of CAA_ACC_12 (S1 Table) using *in silico* fingerprinting (ISIF) (parameters for *in silico* fingerprinting using ISIF software are listed in Table D in S1 File) and found that the sequence was a part of the long interspersed nuclear element (LINE) L1MEf (family L1) on chromosome Y; therefore, the fragment was present only in males.

**Table 3:** Sequences of AFLP markers obtained through NGS and their mapping on a reference sequence of the human genome.

AFLP marker	Fragment length according to GeneMapper (bp)	Fragment length according to NextGENe (bp)	Sequence	Chr.	GRCh38.p7		Gene	RepeatMasker
					**Start**	**End**		
CAG_ACC_32	188	189	TAACAGCCCTGGGAAAAGAATACACTCCTCGGGGGAGGGGAGCCTCTAAAACGGCCACTCTGGGAGTGTCTGCCTTATGCAGTTGTAGATAGGGATGAAACATGCCCTAGTCTCTGGCAGTGTCCACAGGCTTGCTAGGATTAGGAAATTCCAGCCTGGTG	Y	24468005	24468169		LTR (MER11C)
					22834257	22834421		LTR (MER11B)
					25201939	25202103		LTR (MER11C)
CTT_ACA_57	257	257	TAACTTGAGATACAAATTAGACAGAAGCATTCTCAGGAACTGCTTTGTGATGTGTGCATTCAACTCACGGACTTGAACCTTCCCTTTGAGAGAGTCGGTTTTAGAAACAGTTCTTTTGTAGTATCTGAAATTGGATATTTAGAGCGACTTGAGTCCTATGATAGGAAAGGAATACTCCTCACATAAAAATTGGACAGAAGCATTCTCAGAAACTTCTTCGTGATATGTG	5	46489635	46505924	Centromeric and pericentromeric region of several chromosomes	Satellite (ALR/Alpha)
CAG_AGG_56	263	263	AATTCAGGCCAACCATCAAAAGAGACCAAAAAATGGCACTATATTGCTAAGGGTACGATTCACAATGAAAACAAAACAGCTATAAATGTTTGATGCCCATATTTAGAGTTTATTGATACTTTTGTAAGAAGAGAGATCATACTTCATAGGGAACTACAGAATGTCTCAGTAAGTTAGGCAGGGCTTGTTATAGGATTGGGGTCTGTTATAGGGTTCAAGGACCTGTGGTTTTGCTGT	14	63153077	63153313	Intron of LOC105370531	LINE (L1M7); LTR (MER90)
CAA_ACC_41	172	172	AATTCACCAAACACACATCCTCTTTAAGGGCCAGTCAGGCTCTTTGACTGGTAATACTTGACCTCATTTGTACCTTGTCTTCAAACTGCAGCAGAGTTCAGATCCCTGATGCACAAAGCAATGCTCTTTTCACTTCTCCTTGTTGT	18	37996672	37996819		
CAC_ACC_25	152	152	TAACACCAAGAGTGAGCCCTGGCAGGACATTATGGCTCATAATTGTAATCCCAGCACCATGGGAGACTGAGGCGGGCAGATCACTTGAGGTCAGGAGTTTGAGACCAGCCTGGCCAACATGGTG	1	1106045335	106045461		LINE (L1MC1); SINE (AluSz)
CAC_AAG_063	195	196	TAACACAAGGCAACAAGTCACAGGTGAAAATTGAGCCTCTGAGGCCCTGCTGATTGCACAGGGGGGGAAGGCATTTGCATCTCTTCTCACCACAGAGGGGTGGCAGCCAATTACCTCCAGGATTAGAAAAAAGTTGCAAACCTGAGTCTATCTTCACTGACGGGCTTG	19	29062605	29062775		DNA repeat elements (OldhAT1)
CAA_AAC_50	242	240	AATTCAACAAGAGGATGCCTTCTCCTTTTGTCATGCTCAATGATACATTTAGGGTGCCTATTACATGTAGTGAAAATACTCATACAAGTTTTAGGAAAAATCCCCAGACTCAGTTACACAAACATAAACTACCTTACTCATCAAAATAAAACAAAACAAAAACAACAACAGTGAAAACTCTGAAAAGCATCATTCTGCCAAGATAATCACTTGT	5	144671809	144672028		Simple repeat [(ACACAA)n]; Low complexity (A-rich)
CAA_ACC_19	104	105	AATTCACCAATGAAGCCATCAGGTCTAAGGCTTTTCTTTGTCAGGAAGATTTTATTATTGATTCAGTCTCTTTACTTGT	22	23820596	23820676	Intron of SMARCB1	LINE (L1MB3)
CAT_ACC_54	174	175	AATTCACCAGCAACCAGAGGGGAGGGCTGGAGGGAAGATTCGGAGGGGCTGGCCCAAGAGCACCTATGAGCCCCTTTGGAAACCATCCCAATAGGGGAAGCTGGGGGACTTTTCTGGAATAAGGGGGAAGGTTCATGCTATAATCATGT	10	113671332	113671482		

### Characterization of AFLP fragments sequenced through NGS

Generally, several phenomena lead to the presence/absence of a fragment: (a) the presence of a SNP in the MseI or EcoRI restriction site, resulting in introduction/loss; (b) the presence of a SNP in a selective bases of primers; (c) the presence of an insertion/deletion in the sequence; (d) a SNP outside of the restriction sites or the selective bases of primers, resulting in a new restriction site; or (e) a combination or multiplication of (a)—(d). In the next section, the characterization of the sequenced AFLP markers will be presented.

**Fig 3:**
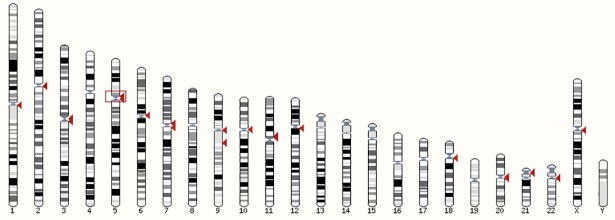
High-scoring segment pair distribution of the CTT_ACA_57 sequence in the human genome. The sequence of CTT_ACA_57 is a part of the variable satellite sequence ALR/Alpha, occurring in the centromeric and pericentromeric regions of several chromosomes. A red box on chromosome 5 represents the best hit.

The NGS-characterized sequence of the AFLP marker CTT_ACA_57 (Table 3), which was significantly associated with the resistance to imatinib (Table 2), was most frequently aligned against Human GRCh38 to chromosome 5 via BLAT. The high-scoring segment pair (HSP) distribution in the human genome provided by Ensembl is shown in Fig 3. Since the sequence is part of the variable satellite sequence ALR/Alpha, occurring in the centromeric and pericentromeric regions of several chromosomes, it was not possible to design specific primers for Sanger sequencing and, thus, to map this region in detail. To confirm the results of NGS, extensive *in silico* analyses were performed. Among 4 obtained sequences (S1 Table), only one insertion/deletion with a very low MAF - = 0.0024/12 and no known SNPs in the restriction sites and/or in selective bases of the MseI and EcoRI primers was identified using BLAT/BLAST databases. Thus, we simulated the presence of a SNP at restriction sites or selective bases of the MseI and EcoRI primers that might lead to the formation of the fragment *in silico* (S2 Table). We identified two SNPs at the MseI restriction site and 3 SNPs in selective bases of the primers; however, they all occurred very rarely, or their MAF was not available, and the CTT_ACA_57 AFLP marker is therefore likely the result of a SNP outside of the restriction site or the selective bases of the primers, or a combination of factors, as noted above.

### Validation of sequences and identified polymorphisms through *in silico* analyses and Sanger sequencing

To analyze the NGS data, we performed AFLP *in silico* analysis of 9 AFLP markers to predict all possible AFLP fragments generated within the range of the fragment size ± 5 bp. S1 and S2 Tables show the sequences obtained through AFLP *in silico* analysis that were used as reference sequences to generate NGS data alignments, to identify all fragment sequences present in the analyzed samples. Using open-source public databases (Ensembl release 80—May 2015; NCBI; UCSC Genome Browser [25]; Dfam 1.4 (May 2015, 1306 entries) [26]; RepeatMasker 4.0.5 [27]), the sequences were then aligned with the human genome, and sequence variants responsible for the introduction or loss of restriction sites were identified (S1 and S2 Tables). We also characterized insertions/deletions that caused changes in the size of a fragment. The sequences obtained through both approaches (AFLP *in silico* and NGS) were confirmed through Sanger sequencing with the designed primers (Table E in S1 File).

The sequence of the CAG_AGG_56 fragment (Table 3), more frequently present in samples of resistant patients, (Table 2), is a part of both LINE L1M7 (family L1) and LTR MER90 (family ERV1). Using Sanger sequencing, we determined that homozygous or heterozygous deletion of two nucleotides (AA) within the sequence of the fragment (rs3047701, MAF - = 0.4034/2020) among the analyzed patients and healthy controls was responsible for the presence of a 263-bp fragment. However, in several samples, the 263-bp fragment was visible in the spectra even though we did not detect deletion of (AA). We suggest that a fragment of the same size probably co-migrated with CAG_AGG_56. Despite the fact that we designed primers (Table E in S1 File) for Sanger sequencing of two other sequences, aligned to chromosome 9 and 16, and predicted by ISIF (S1 and S2 Tables), we did not succeed in revealing the sequence of a co-migrating peak. Based on the results of Sanger sequencing, the presence of a fragment with genotype (AA) was present in 40% of patients who were sensitive to the treatment but was present only in 12.5% of the resistant patients. This finding was not statistically significant for any studied group, probably due to the low number of cases (Table 4).

**Table 4:** The frequencies of presence and statistical significance of AFLP markers after Sanger sequencing.

AFLP marker	Number of detected (1) or undected (0) fragments in all individuals analyzed	Probability that the fragment will be present or absent in the specific cohort of individuals			
**Response to IM**		**Patients sensitive to IM**	**P =**	**Patients resistant to IM**	**P =**
CAG_AGG_56	1 (N = 17)	14	0.1900	3	0.1120
	0 (N = 42)	21		21	
**CML**		**Controls**	**P =**	**Patients**	**P =**
CAC_ACC_25	1 (N = 27)	9	0.2140	18	0.2170
	0 (N = 94)	51		43	
CAC_AAG_063	1 (N = 65)	28	0.0910	37	0.0720
	0 (N = 48)	32		16	
CAT_ACC_55	1 (N = 111)	56	0.6560	55	0.5030
	0 (N = 11)	4		7	

Note: Data were evaluated from the results of Sanger sequencing of 65 CML patients and 60 healthy donors.

In the NGS-identified sequence of CAC_ACC_25 (Table 3), located on chromosome 1, we detected the SNP rs113864098 (C/T). Using Sanger sequencing, we found that a substitution (C/T) resulted in the introduction of an EcoRI restriction site and, thus, the presence of the AFLP marker. The frequency of the fragment was 2-fold higher in patients (30%) than in healthy donors (15%) (Table 4). The sequence of the AFLP marker constituted part of L1MC1 and the AluSz repetition and occurred in an intron of the uncharacterized gene LOC105378886, which is affiliated with the ncRNA class (http://www.genecards.org/cgi-bin/carddisp.pl?gene=LOC105378886). Moreover, using Sanger sequencing, we identified 3 SNPs (rs12082981 A/G; rs6679076 A/G; rs77461468 G/A) that were in linkage disequilibrium with rs113864098 (C/T) and were always present when rs113864098 occurred. rs113864098 and rs12082981 were located within the marker sequence, while rs6679076 and rs77461468 were located 46 bp and 246 bp downstream of the newly introduced EcoRI restriction site, respectively. In the samples from healthy donors, the results of Sanger sequencing were fully in accordance with the fragment analysis. However, in 18/38 patients in whom the fragment was visible in the spectra, Sanger sequencing did not reveal any SNP responsible for the introduction of an EcoRI restriction site. We suggest that in these samples, the fragment of the same length as CAC_ACC_25 that was visible in the spectra was likely the result of size homoplasy [31].

**Fig 4:**
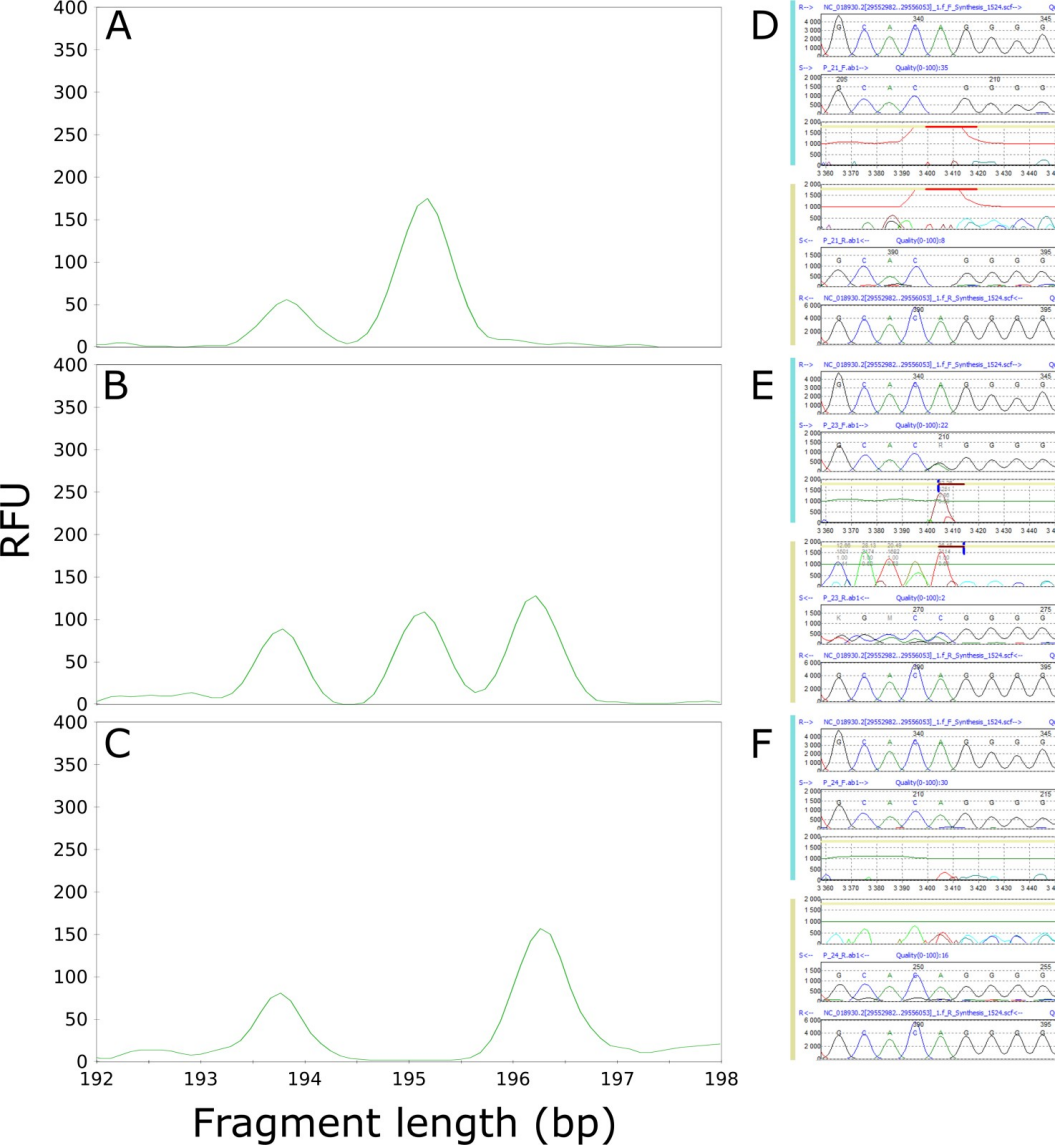
Three different situations regarding the presence/absence of rs11428156 (-/A/G/GG) in the AFLP marker CAC_AAG_063. The presence/absence of rs11428156 (-/A/G/GG) was analyzed in the spectra obtained from fragment analysis and the sequence obtained through Sanger sequencing. Panels A and B show the presence of a 195-bp fragment resulting from the homozygous or heterozygous deletion of adenine, respectively, whereas panel C shows the wild-type sample. Panels D, E, and F show a portion of the sequences with the rs11428156 SNP, evaluated using the software Mutation Surveyor v3.10 (Softgenetics, State College, PA, USA), corresponding to the fragment analysis spectra. RFU—relative fluorescence unit.

Based on the results of Sanger sequencing, we determined that the presence/absence of the CAC_AAG_063 fragment (Table 4) was not dependent on the introduction/loss of a restriction site, but on the presence of the SNPs rs11428156 (-/A/G/GG; MAF - = 0.2015/1009) and rs398120931 (-/G; MAF not available), which were responsible for the observed changes in AFLP marker lengths (Fig 4). The sequence of CAC_AAG_063 constituted part of the DNA transposon OldhAT1 (hAT-Ac family; 2848 bp), located on chromosome 19. The fragment (195 bp) occurred when homozygous or heterozygous deletion of (A; rs11428156) or heterozygous deletion of (G; rs398120931) was detected. These findings were clearly in accordance with the results of the fragment analyses in all patients, although when the sample was wild type and no deletion was present in healthy donors, an artificial fragment that likely originated from the separation of fragments through capillary electrophoresis was observed. The neighboring fragment (196 bp) occurred in different situations when the deletion of (A; rs11428156) or (G; rs398120931) was heterozygous, or a homozygous (A/G) substitution (rs11428156) occurred simultaneously with heterozygous deletion of (G; rs398120931). The frequency of the absence of this 196-bp long fragment was nearly 2-fold higher in patients (13.1%) than in healthy donors (6.7%) (Table 4).

**Fig 5:**
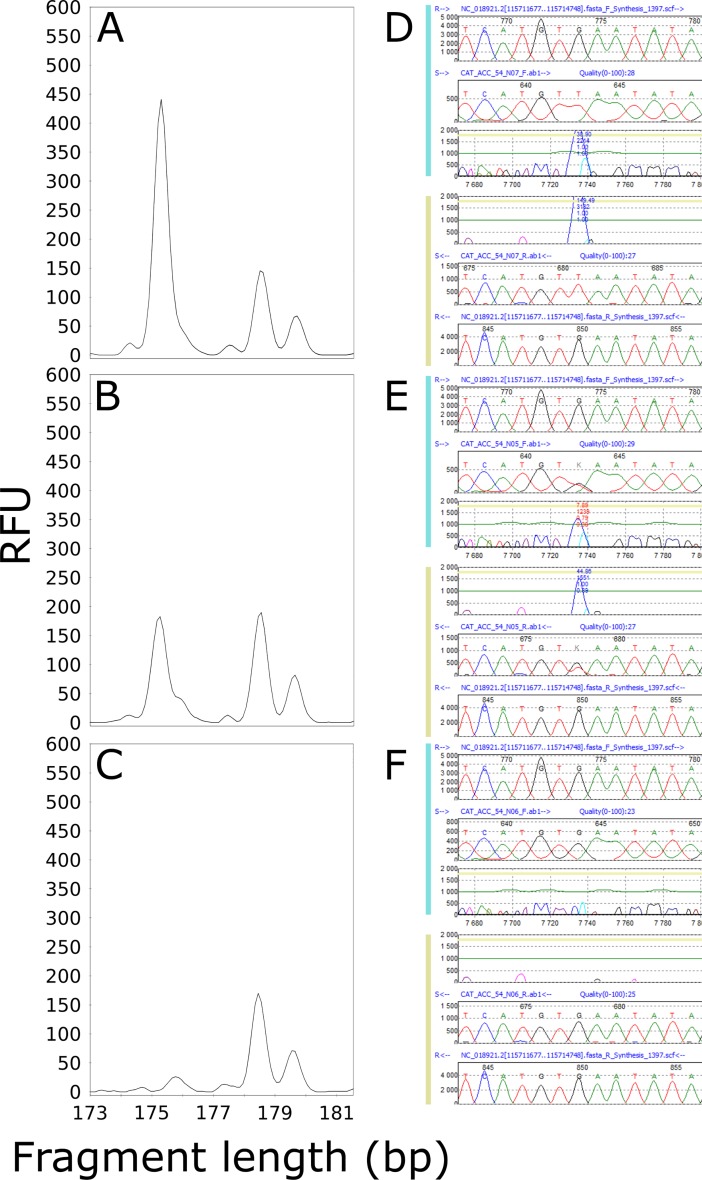
The (G/T) substitution of SNP rs7906704 resulted in the introduction of an MseI restriction site, responsible for the formation of the CAT_ACC_55 fragment (175 bp). The fragment is present (A, B) if the substitution is homozygous (D) or heterozygous (E). Panels C and F illustrate the absence of the fragment, as SNP rs7906704 was not detected. RFU—relative fluorescence unit.

Using NGS, we obtained a sequence that was 1 bp longer than the expected length according to the fragment analysis spectra for the AFLP marker CAT_ACC_54 (Fig 5, Table 3). However, comparison of the results of Sanger sequencing with the electropherogram after fragment analysis revealed that the 175-bp-long sequence (according to NGS) located on chromosome 10 between the NRAP and CASP7 genes corresponded to the neighboring CAT_ACC_55 fragment (Fig 5), which was not statistically significant after evaluation of the fragment analysis results (P = 0.0618; Table 2). Through *in silico* simulation of SNPs at restriction sites (S2 Table), we found that the CAT_ACC_55 fragment formed as a result of the SNP rs7906704 (G/T), leading to the introduction of a new MseI restriction site. The results of Sanger sequencing fully corresponded to the results of the fragment analysis in healthy donors (Table 4); however, in patients, the presence of the one-bp-shorter fragment CAT_ACC_54 impeded scoring and caused misrepresentation of the statistics after fragment analysis (Figure G and Table 2). Neither *in silico* prediction of the sequence (S1 Table) nor simulation of the presence of the SNP in the restriction sites or selective bases of the EcoRI and MseI primers (S2 Table) revealed the sequence of CAT_ACC_54.

## Discussion

In this work, we established an AFLP-AFLP *in silico*-NGS approach enabling screening of the genomes of patients with chronic myeloid leukemia and healthy donors for comparison, and we identified, characterized and mapped loci related to the disease or the response to treatment. We applied AFLP analysis, designed primarily for studies on plant and bacterial genomes, to human genomes isolated from cancer cells. We focused on comparing the DNA fingerprints of the white blood cells of patients with CP-CML against those of healthy donors. To perform the AFLP and AFLP *in silico* analyses, we used two restriction endonucleases: MseI, as a frequently cutting enzyme, and EcoRI, as a rare cutter; the mean distances between the cleavage sites of these enzymes were 150 bp and 3,700 bp, respectively. Sequences recognized by the MseI restriction endonuclease often lie in AT-rich regions. For both enzymes, the longest uncut segments occur on chromosome 6, due to its high content of repeats (http://tools.neb.com/~posfai/TheoFrag/TheoreticalDigest.human.html). Prochazka et al. [23] estimated that 64 primer combinations would provide one AFLP marker every 10–15 cM. Considering all of these factors together, the use of 51 combinations of primers for the selective amplification of products, providing 77 fragments per primer combination on average, ensured equal coverage of the whole human genome.

Although AFLP is a rather dominant marker system, its potential to reveal co-dominant markers, especially in regions with repetitive DNA, including transposons, is unquestionable according to our results. Co-dominant markers enable the genetic patterns of homozygotes and heterozygotes to be distinguished based on the intensity of the signal in a spectrum (whether a locus is homozygous or heterozygous), whereas a dominant marker only provides information about whether an allele is present. As an example of the characterization of a dominant marker, the CAC_ACC_32 fragment (188 bp) was analyzed through AFLP *in silico* as well as NGS, resulting in the same sequence associated with chromosome Y.

Fragment CAC_AAG_063 (195 bp) represents a co-dominant marker that occurred based on indels responsible for a change in AFLP marker length: the SNPs rs11428156 (-/A/G/GG) and rs398120931 (-/G). The sequence of CAC_AAG_063 is a part of the DNA transposon OldhAT1, which was recently shown to be one of the transposable families with the highest proportion of instances of conserved expression between human and chimpanzee induced pluripotent stem cells [32]; however, no active form of the transposon has been found thus far. The difference in the frequency of SNPs indicated by the AFLP markers CAC_AAG_063 (OldhAT1) and CAG_AGG_56 (LINE, LTR) between healthy donors and patients, or between optimally responding patients and resistant patients in our work may support the potential role of repetitive elements in hematological malignancy [33].

The sequence of CTT_ACA_57, an AFLP marker more frequently absent in samples of resistant patient, was not exactly aligned with the human reference genome GRCh38.p7, but was most frequently mapped to chromosome 5 as part of the variable satellite sequence ALR/Alpha. This satellite sequence, occurring in the centromeric and pericentromeric regions of several chromosomes, provides additional evidence that the function of DNA repeats is not well understood and repetitive elements may be part of event-prone regions (recombinations, mutations, breaks). Moreover, Kim et al. [34] reported high-frequency aberrations in ALR/Alpha satellite sequences on chromosomes 2, 10 and 17 in the chronic lymphocytic leukemia (CLL) genome. Taken together, these results may indicate a role of DNA repeats in leukemia etiology.

Based on several studies applying AFLP, the most discussed problem with larger genomes (genome sizes: *Homo sapiens* 3.3 Gb, *C*. *Elegans* 97 Mb, *B*. *subtilis* 4.2 Mb, *Arabidopsis thaliana* 135 Mb) is the co-migration of non-identical AFLP fragments, known as size homoplasy, producing biases in the scoring and interpretation of the results [31,35–37]. It has been predicted that the distribution of AFLP fragments is shifted toward the formation of smaller fragments, increasing the probability of fragments being homoplasious [31,38]. Unfortunately, a positive correlation exists between genome size and homoplasy; however, very little is known about homoplasy in the human genome. Caballero et Quesada [39] noted that the proportion of homoplasy associated with the number of bands generated by a primer combination is very similar for all tested species, including *Homo sapiens*. We observed homoplasy when evaluating the CAG_AGG_56 fragment, in a few samples from patients and healthy donors and in several samples from patients with the CAC_ACC_25 fragment. The co-migrating fragment may have originated from an insertion/deletion, SNP, or somatic mutation anywhere in the genome, or a combination thereof.

The advantages of *in silico* analyses include the generation of virtual fingerprints of genomic DNA and, particularly, savings in terms of time and expenses related to extensive experiments when designing studies. The limitations of AFLP *in silico* lie in the application of such analyses only to those parts of the human genome that have been sequenced with annotated loci. Since the DNA cutting in these analyses is only theoretical, the number of truly amplified fragments that would be detected after separation through capillary electrophoresis is not precisely known. In the established AFLP-NGS approach, we indeed revealed the presence of SNPs/indels leading to the introduction of a new restriction site inside the sequence between two selective bases of EcoRI and MseI primers as well as the presence of more than one SNP or indel in restriction sites or selective bases of primers.

When evaluating data using public databases, there is a need to be cautious when considering SNPs as responsible for the presence/absence of a fragment in samples from “patients”, since its frequency in the “patient” genome could be different from that in the populations sequenced by the 1000 Genomes Project [28] or ExAC [29], and the SNP/indel could therefore occur with a higher/lower frequency than indicated in the databases [40].

## Conclusions

In conclusion, the combination of AFLP together with *in silico* simulation and characterization through next-generation sequencing represents a useful fingerprinting method that can be applied as a smart alternative to microarrays and the still relatively expensive and bioinformatically challenging whole-genome sequencing techniques. The evaluation of data generated from the whole-genome sequencing of hundreds of individual genomes is still nearly impossible to achieve within one or two years of a bioinformatic analysis. On the other hand, AFLP analysis, including evaluation steps, is quick and easy to perform, enabling the choice of specific fragments associated with a studied disease or parameters specific to the analyzed group of individuals. The pre-selected number of fragments that are further processed through NGS markedly reduces the amount of data to be bioinformatically evaluated. One of the unquestionable advantages of the established procedure is its application for the association of variable regions of the human genome with diseases, which remains challenging using whole-genome sequencing technologies.

## Supporting information

S1 File Supporting information including Tables A-E and Figures A-G.
(PDF)

S1 Table Sequences of AFLP markers predicted by ISIF. The analyses were performed in a range of expected fragment length ± 5 bp. Light blue indicates the sequence of CAA_ACC_12 obtained by ISIF that was in accordance with the spectra after the fragment analysis. Light orange indicates sequences obtained by ISIF, NGS and confirmed by Sanger Sequencing. Minor allele frequencies (MAF) of SNPs originated from sequencing projects 1000 Genomes Project (1000 Genomes) [9], TOPMED or the Exome Aggregation Consortium (ExAC) [10].
(XLSX)

S2 Table Simulation of the presence of SNP. Sequences of AFLP markers CTT_ACA_57, CAA_ACC_41, CAT_ACC_54, CAT_ACC_55, CAC_ACC_25, CAG_AGG_56, and CAA_AAC_50 after a simulation of the presence of SNP in restriction sites or selective parts of EcoRI and MseI primers by ISIF. The analyses were performed in a range of the expected fragment length ± 5 bp. Light green indicates sequences obtained by ISIF after simulation of SNP, NGS and confirmed by Sanger sequencing. Minor allele frequencies (MAF) of SNPs originated from sequencing projects 1000 Genomes Project (1000 Genomes) [9], TOPMED or the Exome Aggregation Consortium (ExAC) [10].
(XLSX)
