# Supplementary material for: AFLP-AFLP in silico-NGS approach reveals polymorphisms in repetitive elements in the malignant genome
Source: PLoS One. 2018 Nov 8;13(11):e0206620. doi: 10.1371/journal.pone.0206620 (PMC6224067; doi:10.1371/journal.pone.0206620)
Supplement: S1 File — Supporting information including Tables A-E and Figures A-G. (PDF) [file pone.0206620.s001.pdf]

Supporting information

Chronic myeloid leukemia

Chronic myeloid leukemia is characterized by reciprocal translocation between chromosomes 9 and 22 t(9;22)(q34;q11), known as the Philadelphia chromosome, resulting in a BCR-ABL1 fusion oncogene. BCR-ABL1 encodes a chimeric tyrosine kinase, which is the key protein with an unregulated function resulting in neoplastic hematopoiesis and CML. It has been suggested that the first hit leading to CML development is a mutagenic event leading to the transformation of a normal hematopoietic stem cell to a premalignant progenitor cell population and subsequent generation of the BCR-ABL1 oncogene [1]. However, the mechanism underlying the formation of the Philadelphia chromosome and clonal expansion of pre-CML progenitor cells is still unknown [2-4]. The development of tyrosine kinase inhibitors (TKIs), such as imatinib, inhibiting the activity of the BCR-ABL1 tyrosine kinase, represented a significant breakthrough in CML treatment. The overall survival of patients treated with imatinib longer than 5 years is between 83 % and 97 %; however, 20-30 % of patients are resistant to this therapy, resulting in therapeutic failure [5]. Fortunately, second- and third-generation TKIs, such as nilotinib, dasatinib, bosutinib, and ponatinib, have been successfully introduced in the treatment of CML and have been efficient in overcoming imatinib failure. One of the best-studied mechanisms of TKI resistance is the development of point mutations in the kinase domain of the BCR-ABL1 fusion gene. Other mechanisms of resistance, such as amplification of the BCR-ABL1 gene [3], loss of kinase target dependence [4], and pharmacogenomics, are still a subject of interest and remain to be elucidated.

Patient characteristics

The median age of the patients was 55 years (range 18-84 years), including 25 women (38 %) and 40 men (62 %). According to the recommendations of the European LeukemiaNet (ELN), the patients were divided into two groups based on their response to imatinib treatment at 12 months after therapy initiation [5]. Patients with optimal responses to imatinib therapy (N=39) achieved a major molecular response after 12 months (MMR; BCR-ABL1^{IS} ≤ 0.1 %; IS = International Scale). The second group consisted of patients exhibiting treatment failure, or patients in the "Warning" category (N=26). At this time, treatment failure was not associated with the development of resistance to imatinib due to mutation in the kinase domain of BCR-ABL1, except in one patient in whom a T315I mutation was detected through Sanger sequencing at 9 months after therapy initiation (4 patients developed mutations after 12 months of imatinib treatment). All 65 patients were treated with the standard daily dose of imatinib (400 mg/day) for 12 months after therapy initiation. Patient characteristics are summarized in Table A.

Table A. Patient characteristics.

ID	LYZ	SEX	AGD	SMO	W	H	KPS	ECO	CS	PSS	WBC	BLA	HR	SOS	ES	TYP	PCR	RES	NEM	NPM	ACA	PHPM
P_03	trizol	M	52	non-smoker	73	176	90	1	yes	2	332.22	0.5	intermediate risk	0.74	29	b3a2	53	1	0	n.a.	n.a.	n.a.
P_04	trizol	F	63	non-smoker	71	163	90	1	no	n.a.	43.95	0	intermediate risk	n.a.	n.a.	b3a2	74	0	10	10	no	100
P_05	trizol	M	50	current smoker	108	171	90	1	yes	n.a.	79.9	0.5	low risk	n.a.	n.a.	b3a2	50	1	9	9	no	100
P_06	trizol	M	55	current smoker	79	186	100	0	yes	20	9.39	1	n.a.	1.7	155.6	b2a2	37	0	9	8	no	88.89
P_07	trizol	M	37	non-smoker	97	186	100	0	no	0	21.42	0	low risk	n.a.	n.a.	b2a2	55	1	n.a.	n.a.	yes	100
P_08	trizol	F	50	non-smoker	80	170	90	1	yes	15	336.36	1.5	intermediate risk	1.22	70.5	b2a2	184	0	10	9	no	90
P_09	ITG	F	62	current smoker	82	163	90	1	yes	4	387.2	1	high risk	0.97	89.5	b3a2	264	0	12	12	no	100
P_10	ITG	M	61	ex-smoker	77	178	100	0	no	0	78.77	1	intermediate risk	0.79	35	b3a2	68	1	8	8	no	100
P_11	ITG	M	56	non-smoker	160	190	100	0	no	0	79.58	0.5	low risk	0.71	17.5	b3a2	158	0	6	6	no	100
P_12	ITG	M	56	non-smoker	73	172	90	1	yes	0	85.76	0.5	low risk	0.81	13.3	b2a2	124	0	13	13	no	100
P_13	ITG	F	46	ex-smoker	70	165	100	0	yes	0	46.1	0	low risk	0.6	23.1	b2a2	61	1	23	23	no	100
P_14	ITG	F	67	current smoker	94	172	100	0	yes	0	56.11	1	intermediate risk	0.91	35	b3a2	52	1	0	n.a.	n.a.	n.a.
P_15	ITG	M	54	non-smoker	64	177	90	1	yes	12	272.14	3	high risk	1.32	111	b2a2	135	1	22	22	no	100
P_16	trizol	M	84	non-smoker	69	170	100	0	no	0	22.46	0	intermediate risk	0.97	53.2	b3a2	44	1	14	10	no	71.43
P_17	trizol	F	57	ex-smoker	96	175	100	0	no	0	32.79	0	low risk	0.68	14	b3a2	32	1	22	21	no	95.45

P_19	trizol	F	55	non-smoker	72	151	90	1	yes	0	163.39	1.5	intermediate risk	0.79	21	b3a2	109	1	0	n.a.	n.a.	n.a.
P_20	trizol	M	69	non-smoker	87	173	100	0	no	0	36.7	0	intermediate risk	0.82	21	b3a2	46	1	13	8	no	61.54
P_21	trizol	F	50	non-smoker	68	162	90	1	yes	15	46.84	4.7	high risk	1.71	139.1	b3a2	99	0	0	n.a.	n.a.	n.a.
P_23	trizol	M	44	ex-smoker	78	180	100	0	no	0	22.44	0	low risk	0.65	77	b3a2	33	1	2	n.a.	no	n.a.
P_24	trizol	F	18	non-smoker	65	169	100	0	yes	4	268.67	2.5	low risk	0.78	54.5	b2a2	25	0	0	n.a.	n.a.	n.a.
P_25	trizol	F	68	non-smoker	115	170	80	2	no	0	42.35	1	intermediate risk	0.93	86.8	b3a2	20	0	22	18	no	81.82
P_26	trizol	F	46	non-smoker	79	175	90	1	yes	20	313.17	7	high risk	2.21	187.8	b3a2	32	0	9	9	no	100
P_27	ITG	M	80	non-smoker	82	165	90	1	no	2	80	1	intermediate risk	1.06	50	b3a2	51	0	0	n.a.	n.a.	n.a.
P_28	ITG	M	56	non-smoker	87	175	90	1	yes	25	259.76	0.5	high risk	1.66	118.9	b3a2	35	0	16	11	no	68.75
P_30	ITG	M	38	non-smoker	77	177	100	0	no	0	84.39	0	low risk	0.59	84	b3a2	134	1	7	7	no	100
P_31	ITG	F	57	non-smoker	57	160	90	1	yes	8	150.88	5	high risk	1.54	151	b2a2	213	1	12	12	no	100
P_32	ITG	F	69	non-smoker	80	164	100	0	yes	0	67.64	1	low risk	0.85	2.8	b2a2	69	0	12	7	no	58.33
P_33	ITG	M	68	ex-smoker	90	172	100	0	no	0	43.65	1	low risk	0.87	50.4	b3a2	44	1	20	20	no	100
P_34	ITG	M	31	current smoker	92	167	100	0	no	0	45.44	1	low risk	0.6	42	b2a2	57	1	14	14	no	100
P_35	ITG	M	30	non-smoker	72	178	90	1	yes	25	553.47	2.5	high risk	1.48	135	b2a2	133	0	2	2	no	100
P_36	ITG	F	51	non-smoker	68	173	100	0	no	0	151.32	0.8	low risk	0.74	35.7	b2a2	83	1	0	n.a.	n.a.	n.a.
P_37	ITG	F	41	current smoker	52	168	90	1	yes	20	323.56	5	intermediate risk	1.75	115	b2a2	67	1	0	n.a.	n.a.	n.a.
P_38	ITG	M	52	non-smoker	94	172	100	0	no	0	87.24	0	low risk	0.65	0	b2a2	117	1	7	4	no	57.14

P_39	ITG	F	32	non-smoker	60	158	100	0	no	0	29.32	1.5	low risk	0.79	14	b2a2	57	1	22	22	no	100
P_40	ITG	M	70	non-smoker	90	182	100	0	yes	0	107.5	4	intermediate risk	1.13	7	b2a2	204	1	n.a.	n.a.	n.a.	n.a.
P_41	ITG	F	37	non-smoker	75	165	90	1	yes	0	59.51	0	intermediate risk	0.58	56	b2a2	96	0	22	22	no	100
P_42	ITG	M	83	non-smoker	58	172	100	0	no	0	42.26	2	intermediate risk	1.24	70	b3a2	69	0	6	6	no	100
P_43	ITG	M	67	non-smoker	99	185	90	1	no	0	27.5	0	intermediate risk	0.81	70	b3a2	34	1	22	22	no	100
P_44	ITG	M	64	non-smoker	95	190	90	1	no	0	31	1	intermediate risk	0.83	21	b3a2	51	1	22	22	no	100
P_45	ITG	M	55	ex-smoker	98	180	90	1	no	0	22.3	1	intermediate risk	0.8	35	b2a2	144	1	22	22	no	100
P_46	ITG	M	62	current smoker	104	186	90	1	yes	20	308	1	high risk	1.57	87	b2a2	237	0	22	22	no	100
P_47	ITG	M	30	non-smoker	70	170	90	1	yes	16	245.2	1.5	low risk	1.05	102.5	b3a2	93	0	22	22	no	100
P_48	ITG	M	61	ex-smoker	100	176	90	1	no	10	578.87	4	high risk	1.54	103	b3a2	361	0	22	22	no	100
P_49	ITG	F	34	ex-smoker	54	156	100	0	yes	1	84.23	2	low risk	0.68	32	b2a2	183	1	0	n.a.	n.a.	n.a.
P_50	ITG	M	51	ex-smoker	78	169	80	1	yes	0	22	0	intermediate risk	0.74	28	b3a2	88	0	22	22	no	100
P_51	ITG	M	39	non-smoker	95	188	90	1	yes	0	152.55	2	low risk	0.66	14	b3a2	166	0	22	22	no	100
P_52	ITG	M	20	ex-smoker	65	175	100	1	yes	0	188.13	2	low risk	0.53	14	b2a2	50	0	22	20	no	90.91
P_53	ITG	F	49	ex-smoker	49	160	80	2	yes	5	257.42	7.5	intermediate risk	1.82	90	b3a2	131	0	8	8	no	100
P_54	ITG	M	45	current smoker	92	187	100	0	yes	0	55.98	1	low risk	0.65	28	b3a2	49	0	13	13	no	100
P_55	ITG	M	34	ex-smoker	73	182	90	1	no	10	216.67	0.5	low risk	0.79	54	b2a2	421	1	21	20	no	95.24
P_56	ITG	M	56	ex-smoker	117	190	100	0	no	0	50.5	2	intermediate risk	0.82	49	b3a2	47	1	8	8	no	100

P_57	ITG	M	38	current smoker	96	176	100	0	no	1	32.15	2	low risk	0.73	67	b2a2	92	1	22	22	no	100
P_58	ITG	M	47	non-smoker	85	185	100	0	yes	12	262.46	2.5	high risk	1.23	86.5	b3a2	169	1	10	10	no	100
P_59	ITG	M	56	non-smoker	118	199	100	0	yes	0	29.77	0	intermediate risk	0.94	35.7	b3a2	19	1	18	18	no	100
P_60	ITG	M	75	current smoker	81	167	90	1	yes	0	74.6	0	intermediate risk	0.86	7	b2a2	105	1	8	8	no	100
P_63	ITG	M	33	non-smoker	86	185	90	1	yes	14	5.2	0	intermediate risk	0.83	91	b3a2	73	1	22	22	no	100
P_64	ITG	F	61	non-smoker	92	165	100	0	no	0	74.51	0	intermediate risk	0.74	14	b3a2	91	1	5	5	no	100
P_65	ITG	M	69	non-smoker	88	173	90	1	yes	0	55.5	0	intermediate risk	0.8	7	b3a2	58	1	10	10	no	100
P_66	ITG	F	75	non-smoker	85	165	80	2	no	0	15.77	0	high risk	2.26	84	b3a2	16	1	n.a.	n.a.	n.a.	n.a.
P_67	ITG	F	56	non-smoker	64	171	100	0	yes	11	141	2	high risk	1.21	65	b2a2	153	1	5	5	no	100
P_68	ITG	F	55	non-smoker	63	159	90	1	no	0	16.71	0	intermediate risk	0.74	63	b3a2	29	1	22	22	no	100
P_69	ITG	M	77	current smoker	75	166	90	1	yes	1	91	0	intermediate risk	0.98	95	b2a2	153	1	15	15	no	100
P_70	ITG	M	55	non-smoker	85	183	80	2	yes	0	46.37	1	intermediate risk	n.a.	n.a.	b3a2	52	0	22	22	no	100
P_71	ITG	F	51	non-smoker	75	169	80	1	yes	13	252	4	high risk	1.54	n.a.	b3a2	153	0	22	22	no	100
P_72	ITG	F	39	non-smoker	63	167	90	1	yes	0	35	0	low risk	0.64	35	b3a2	126	1	20	20	no	100

n.a. data not available

		abb.

Patient	Sample ID	ID
	trizol/ITG	LYZ
	Male (M)/Female (F)	SEX
Demographics	Age at diagnosis	AGD
	Smoking status	SMO
Physical examination	Weight (kg)	W
	Height (cm)	H
	Karnofsky performance status (%)	KPS
	ECOG/WHO	ECO
	Clinical symptoms and signs of disease	CS
	Palpable spleen size below costal margin (cm)	PSS
Full blood count	WBC (x10^9/l)	WBC
	Blasts (%)	BLA
Prognosis	Hasford risk	HR
	Sokal score	SOS
	EUTOS score	ES
Molecular biology	Type of transcript	TYP
	RQ-RT-PCR results (% IS)	PCR
	Treatment response (0-resistant, 1-responsive)	RES
Cytogenetics	No. of evaluated mitoses	NEM

	No. of Ph positive mitoses	NPM
	Additional chromosomal abnormalities in Ph- positive cells (ACAs)	ACA
	Ph positive mitoses (%)	PHPM

Clinical and molecular genetic parameters of patients in CP-CML at the time of diagnosis and the response to the imatinib treatment.

AFLP analyses

The AFLP technique can be used to detect polymorphisms in DNA when no information about the genome is known. Following restriction enzyme digestion of DNA, a subset of DNA fragments is selected for PCR amplification and separation by capillary electrophoresis. In detail, the whole procedure is shown in Figure A.

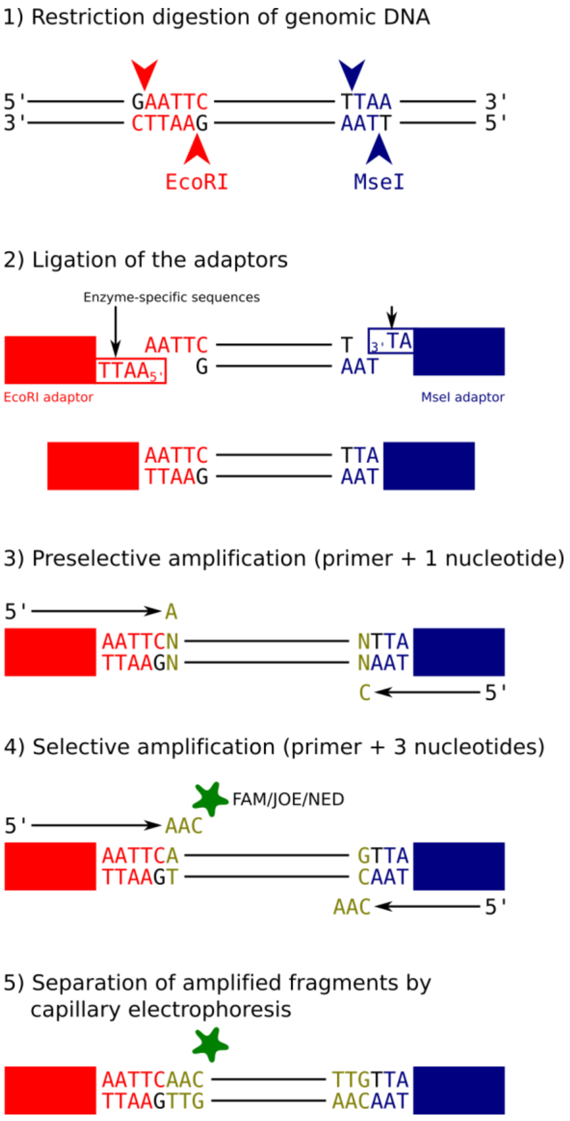

Figure A. Scheme of the AFLP procedure. DNA is digested by two restriction endonucleases at specific sites. Adaptors are then ligated to the ends of the DNA fragments resulting in the template for further amplifications. Selective amplification with an EcoRI and an MseI primer amplifies primarily

EcoRI-MseI-ended fragments. The PCR product is separated by capillary electrophoresis and only the strands that contain the dye-labeled EcoRI are detected.

AFLP is known to be highly reproducible technique for plant genomes. As well, the same results were achieved when applied on the human genome [7]. Despite that, we tested the reproducibility at the two levels. Firstly, we examined subtypes of cells isolated from the peripheral blood (polymorphonuclear cells and mononuclear cells) as the initial biological material for the DNA isolation and compared data with total leukocytes (Figure B). After electrophoretic separation of AFLP fragments, all cell subpopulations provided the identical AFLP fingerprints. Additionally, replicates of samples from different preselective amplifications were tested (Figure C) and separation of AFLP fragments resulted in the same AFLP profiles. Based on these results, we decided to use total leukocytes for DNA isolation and all further AFLP analyses.

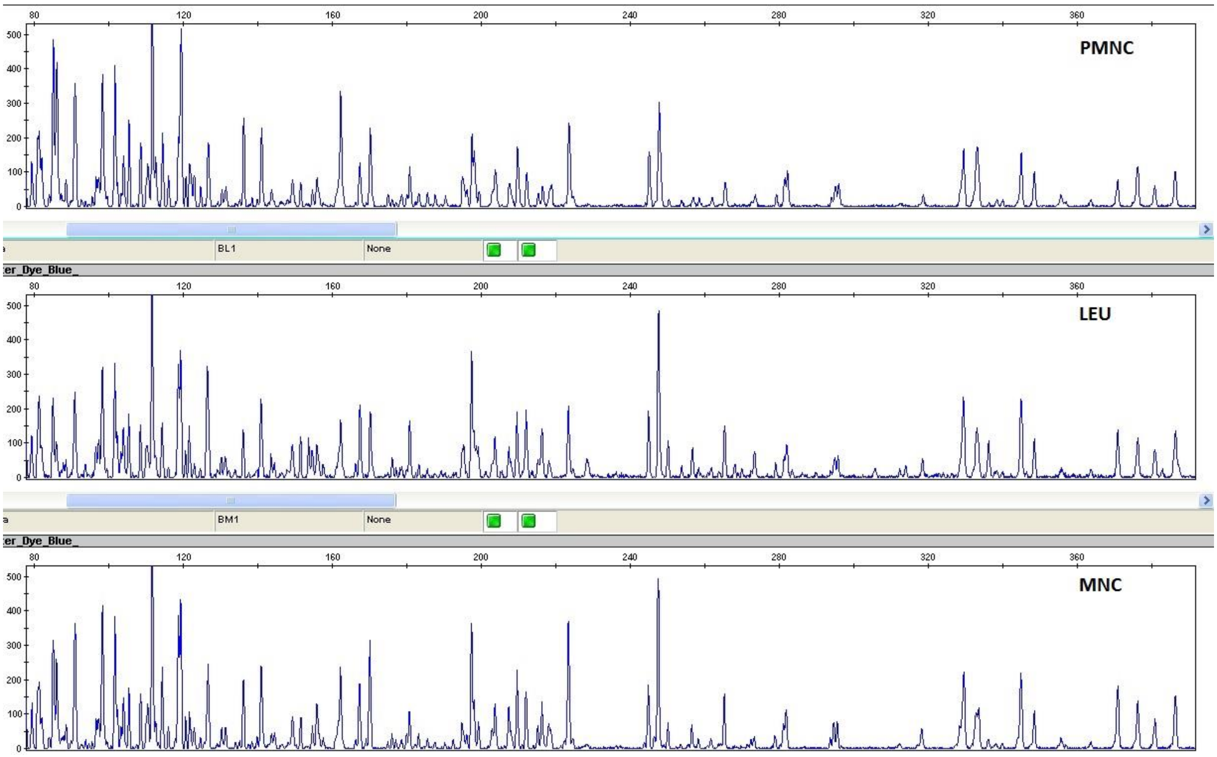

Figure B. Separated subpopulations of leukocytes isolated from peripheral blood provided identical AFLP patterns compared to total leukocytes. Illustrative electropherograms of PCR

products after selective amplification of AFLP fragments with one primer combination in polymorphonuclear cells (PMNC), total leukocytes (LEU) and mononuclear cells (MNC).

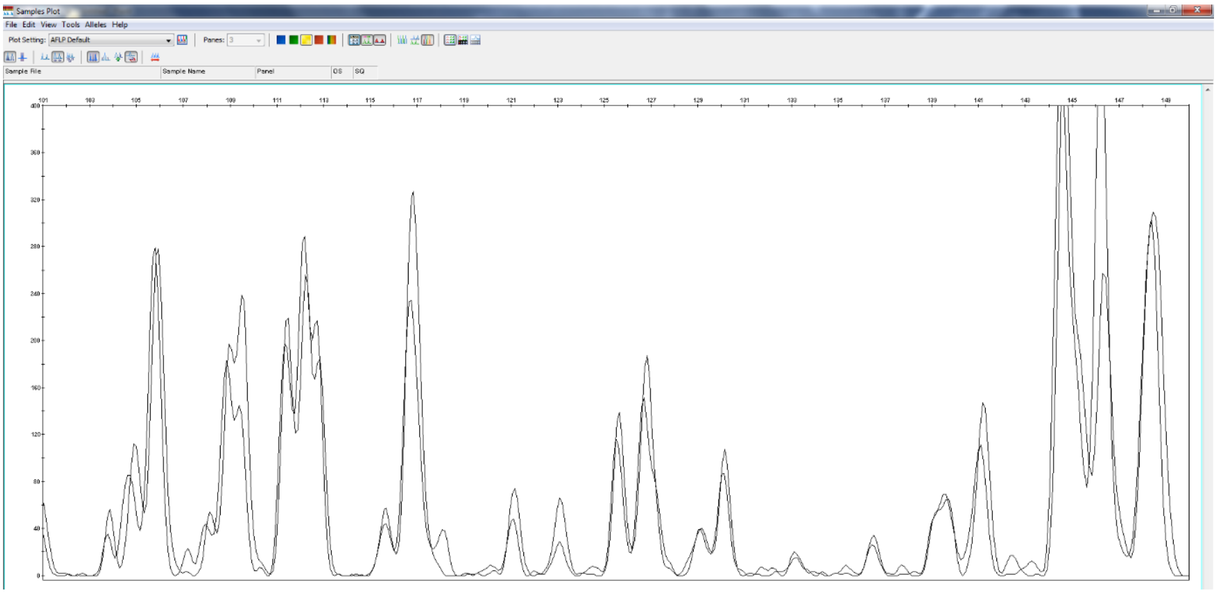

Figure C. Replicates of preselective and selective amplification steps resulted in the identical AFLP pattern. For illustration we show the overlapped electropherograms of PCR products of patients replicate after selective amplification of AFLP fragments with CAC and ACC primer combination.

Among all 64 possible combinations of the EcoRI and MseI primers, 51 (Table 1) were used for selective amplification (Panels A and C represent an ideal AFLP profile), whereas 13 were eliminated based on the small number of DNA fragments (Figure D, Panel B) suitable for scoring as 1 or 0, for the presence and/or absence of the fragment, respectively, and/or a low intensity of the signal (Figure D, Panel D).

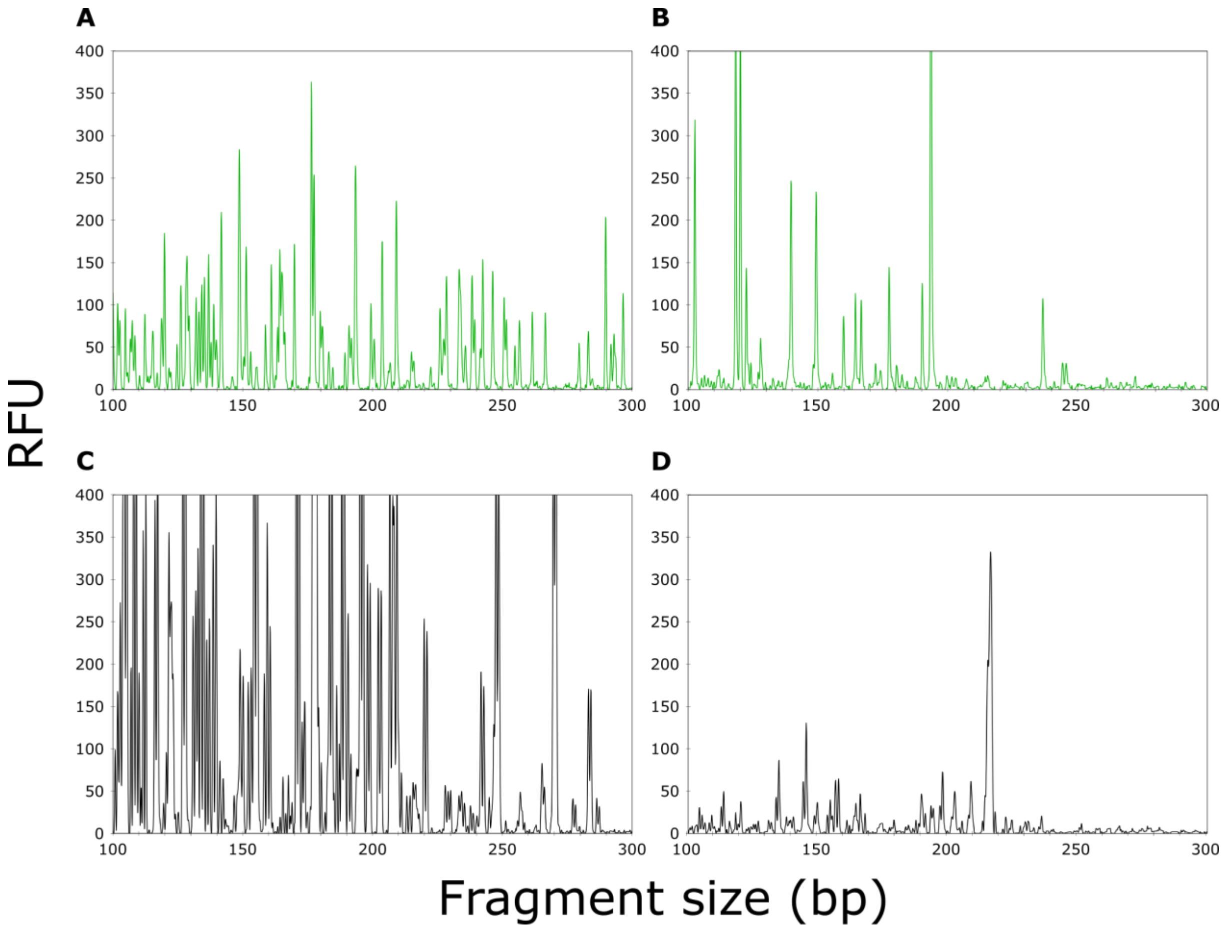

Figure D. The comparison of suitable and unsuitable combination of primers. Electropherograms of AFLP samples amplified using two different primer combinations after separation on an ABI PRISM 3130 Genetic Analyzer. An example of an amplification with the suitable combination of primers (A; CTC_AGG and C; CTC_AGC) is shown in addition to an example of an amplification with the primer combination (B; CTC_AGG and D; CAT_AGC) that was deemed to be unsuitable for selective amplification due to a low number of peaks and/or low signal during fluorescence detection. Green and black correspond to the fluorescent dyes JOE and NED, respectively. RFU – relative fluorescence unit

Preparation of fragments for NGS analysis

The presence of fragments of the required length extracted from agarose gels after electrophoretic separation was confirmed by fragmentation analysis on an ABI PRISM 3130 Genetic Analyzer and evaluated in GeneMapper software v4.1 (Thermo Fisher Scientific), as described in the Materials and Methods. Three gel regions were tested for each fragment (Figure E). The region containing the greatest amount of the particular fragment and the smallest number of other undesired fragments was used for further processing.

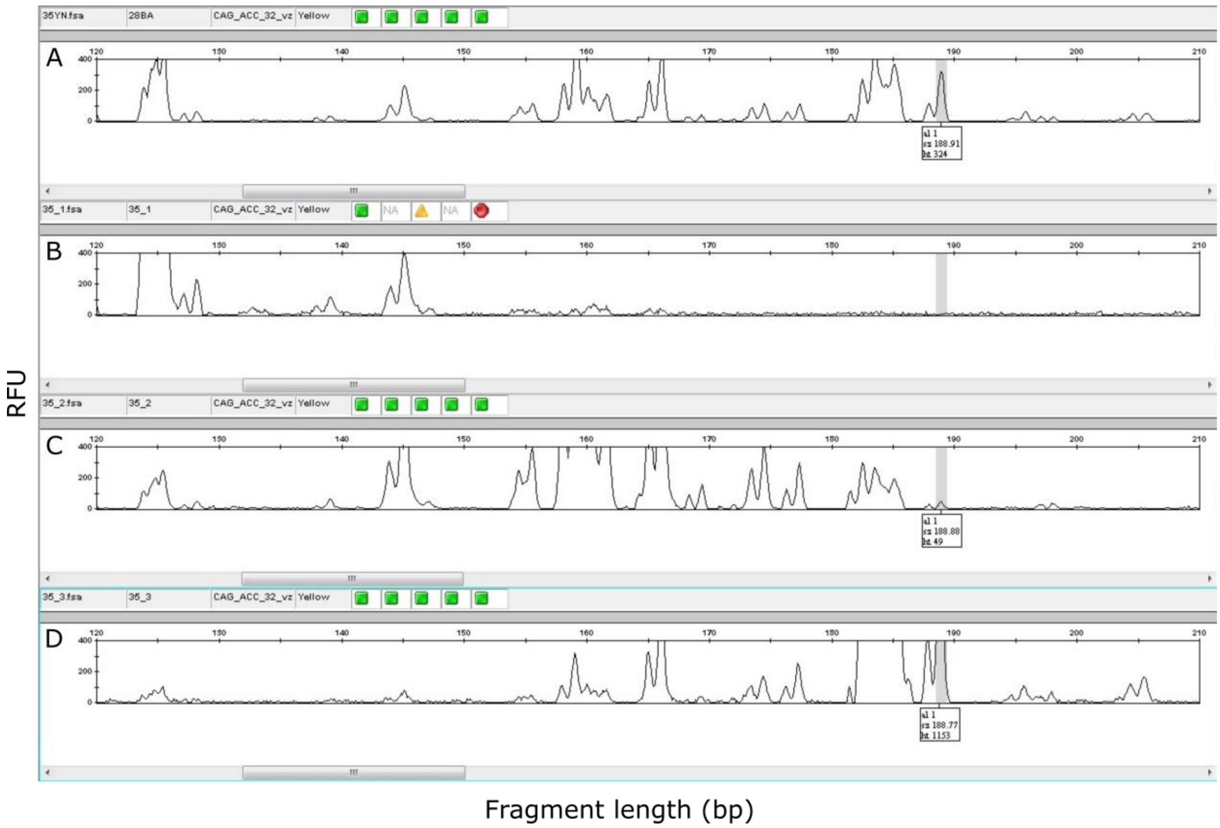

Figure E. Electropherograms of one of the AFLP samples selected for further NGS analysis. An example of fragmentation analysis following the original selective amplification of CAG_ACC_32 (A; size 188 bp) and 3 regions (B, C, D) containing fragments with different sizes extracted from the agarose gel after electrophoretic separation. Region D, containing the largest amount of the

CAG_ACC_32 fragment, was used for further NGS analysis. The CAG_ACC_32 fragment is highlighted in gray.

Preparation of the amplicon library was performed according to the manufacturer's recommendations, with modifications in several steps. To enable the ligation of adaptors with molecular identifier tags (MIDs), the ends of purified DNA fragments were blunted. Based on the results of quantification of amplicons using RT-qPCR, we determined that the adaptors self-ligated at the concentration indicated in the manufacturer's protocol (Roche Applied Science), which resulted in a product in the no template control that was visible after electrophoretic separation. Based on the optimization of adaptor concentrations, assessed using an Agilent High Sensitivity DNA Assay on an Agilent 2100 Bioanalyzer (Agilent Technologies, Santa Clara, CA, USA), a 100x lower concentration than that indicated in the protocol was used for further amplicon library preparation, to reduce the number of self-ligated adaptors.

To clean up the amplicon library of short fragments less than 100 bp in length, we purified the sample using a 1.8:1 ratio of Agencourt AMPure XP beads to the sample (Beckman Coulter), without the Sizing solution (Roche Applied Science), which according to the protocol, removes dsDNA of less than 300 bp and would therefore also remove the AFLP fragments of interest. The fragments were purified only once, since the second purification led to a loss of products. A total of 25 µl of the sample was added to 45 µl of Agencourt AMPure XP beads, followed by vortexing until the mixture was homogeneous. The mixture was then incubated for 10 minutes at room temperature, and the tubes were subsequently placed on a magnetic ring stand and incubated at room temperature until the supernatant was clear. The supernatant was then discarded; 100 µl of 70 % ethanol was added to each sample; and the tubes were placed on the magnetic ring stand. After clearing of the supernatant, ethanol was discarded, and the tubes were left open to vaporize the remaining ethanol. Next, 22 µl of TE buffer was added, and the samples were mixed in a circular motion and incubated for 2 min at room temperature. After clearing of the supernatant of the samples on the magnetic

stand, 20 µl of the supernatant was transferred to a new tube. The presence/absence of self-ligated adaptors and amplified products was assessed using an Agilent High Sensitivity DNA Assay on an Agilent 2100 Bioanalyzer (Figure F), with quantification via RT-qPCR (KAPA Library Quantification Kits; KAPABIOSYSTEMS). The products were then diluted and pooled in an equimolar ratio. Emulsion PCR was performed using the GS Junior Titanium emPCR Kit (Lib-L). Sequencing was performed according to the manufacturer's protocol (Roche Applied Science).

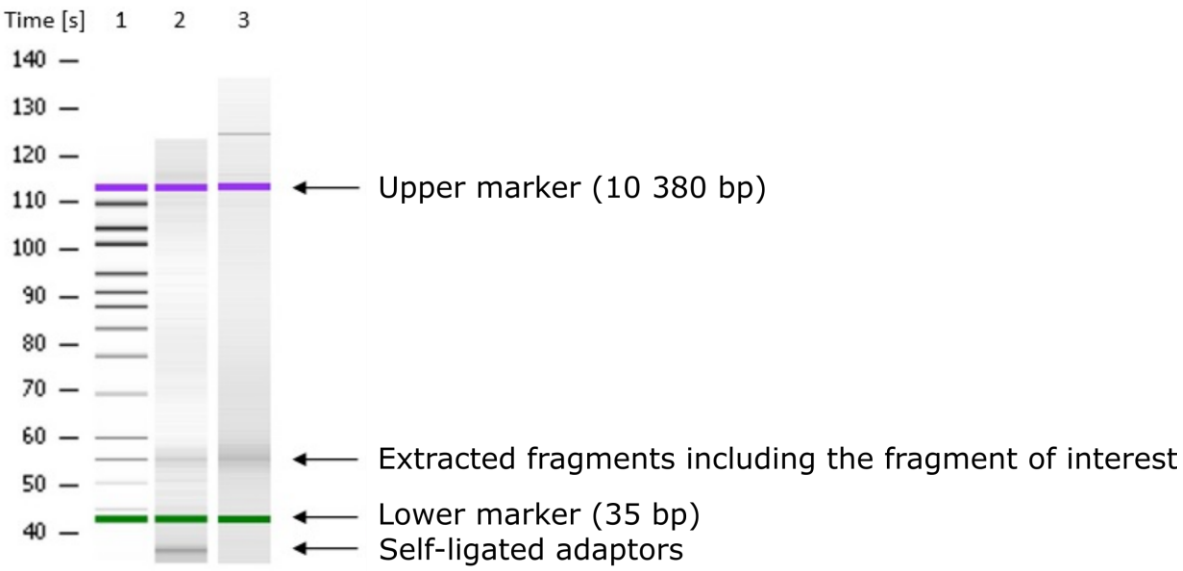

Figure F. Bead purification removed undesired short fragments and self-ligated adaptors. Electropherogram of an amplicon library before (lane 2) and after (lane 3) purification. The removal of short fragments and self-ligated adaptors using Agencourt AMPure XP beads at 1.8x sample volume was assessed with an Agilent High Sensitivity DNA Assay on an Agilent 2100 Bioanalyzer. Lane 1: ladder; lane 2: sample before purification with beads; lane 3: sample after purification with beads.

Results

AFLP fingerprints clearly distinguished patients with CML from healthy individuals

Table B. The set of AFLP markers related to disease (N=180).

AFLP marker	Fragment length (bp)	Number of detected (1) or undetected (0) fragment in all individuals analyzed	Number of detected (1) or undetected (0) fragment in healthy donors	P=	Number of detected (1) or undetected (0) fragment in patients	P=
CAG_ACT_17	135	1 (N=63)	0	1.82E-15	63	2.90E-11
		0 (N=30)	30		0	
CAG_AAC_033	122	1 (N=30)	30	1.82E-15	0	2.90E-11
		0 (N=63)	0		63	
CAG_AAC_051	153	1 (N=30)	30	1.82E-15	0	2.90E-11
		0 (N=63)	0		63	
CAG_AAC_0	205	1 (N=63)	0	1.82E-	63	2.90E-

AFLP marker	Fragment length (bp)	Number of detected (1) or undetected (0) fragment in all individuals analyzed	Number of detected (1) or undetected (0) fragment in healthy donors	P=	Number of detected (1) or undetected (0) fragment in patients	P=
CTT_AAG_070	188	1 (N=66)	30	6.11E-05	36	0.0231
		0 (N=26)	0		26	
CTT_AAG_101	273	1 (N=26)	0	6.11E-05	26	0.0231
		0 (N=66)	30		36	
CTT_ACA_56	247	1 (N=73)	12	6.83E-05	61	0.0012
		0 (N=15)	13		2	
CAC_AAG_0	80	1 (N=13)	13	7.30E-	0	0.000

AFLP marker	Fragment length (bp)	Number of detected (1) or undetected (0) fragment in all individuals analyzed	Number of detected (1) or undetected (0) fragment in healthy donors	P=	Number of detected (1) or undetected (0) fragment in patients	P=
CTG_ACG_37	200	1 (N=51)	8	0.0027	43	0.0318
		0 (N=42)	22		20	
CAC_AGG_70	338	1 (N=48)	7	0.0028	41	0.0428
		0 (N=45)	23		22	
CTT_ACA_49	213	1 (N=20)	0	0.0029	20	0.0976
		0 (N=68)	25		43	
CAG_ACA_0	86	1 (N=75)	30	0.003	45	0.073

74		0 (N=30)	30	15	0	11
CAA_ACA_03	79	1 (N=62)	0	2.51E-15	62	4.79E-11
		0 (N=30)	30		0	
CAA_ACA_22	142	1 (N=30)	30	2.51E-15	0	4.79E-11
		0 (N=62)	0		62	
CAG_ACC_02	79	1 (N=30)	30	3.49E-15	0	3.94E-11
		0 (N=61)	0		61	
CAG_ACT_55	357	1 (N=62)	0	4.86E-15	62	4.03E-10
		0 (N=31)	30		1	
CAA_ACA_23	144	1 (N=61)	0	6.72E-15	61	3.66E-10
		0 (N=31)	30		1	
CAG_AGG_13	107	1 (N=32)	30	1.74E-14	2	3.24E-09
		0 (N=60)	0		60	
CAG_AAC_062	177	1 (N=34)	30	7.76E-14	4	4.61E-08
		0 (N=59)	0		59	
CAG_AAC_065	181	1 (N=55)	0	2.18E-12	55	1.10E-06
		0 (N=38)	30		8	
CAG_AAC_066	182	1 (N=55)	0	2.18E-12	55	1.10E-06
		0 (N=38)	30		8	
CTT_ACA_47	208	1 (N=65)	2	6.66E-12	63	7.72E-09
		0 (N=23)	23		0	
CTC_AAG_68	264	1 (N=57)	1	1.28E-11	56	6.58E-07
		0 (N=35)	29		6	
CAT_ACC_69	199	1 (N=52)	0	2.13E-11	52	5.01E-06
		0 (N=41)	30		11	
05		0 (N=78)	16	05	62	2
CTG_AGG_38	188	1 (N=15)	14	8.78E-05	1	0.0004
		0 (N=78)	16		62	
CTC_AAC_06	89	1 (N=22)	17	1.04E-04	5	0.0017
		0 (N=71)	13		58	
CAT_ACC_89	286	1 (N=18)	16	1.08E-04	2	0.0004
		0 (N=67)	14		53	
CAG_AGG_62	294	1 (N=25)	0	1.11E-04	25	0.0310
		0 (N=67)	30		37	
CAG_AAC_059	168	1 (N=25)	0	1.15E-04	25	0.0318
		0 (N=68)	30		38	
CAG_AGG_40	186	1 (N=40)	3	0.0001	37	0.0143
		0 (N=52)	27		25	
CAG_ACA_67	215	1 (N=74)	15	0.0002	59	0.0019
		0 (N=18)	15		3	
CTC_AAG_57	228	1 (N=74)	15	0.0002	59	0.0019
		0 (N=18)	15		3	
CAT_ACC_54	174	1 (N=24)	0	0.0002	24	0.0306
		0 (N=69)	30		39	
CTT_ACC_32	154	1 (N=24)	0	0.0002	24	0.0192
		0 (N=67)	30		37	
CAG_ACC_39	226	1 (N=24)	0	0.0002	24	0.0192
		0 (N=67)	30		37	
CAG_ACC_46	252	1 (N=24)	0	0.0002	24	0.0192
		0 (N=67)	30		37	
7		0 (N=17)	0	7	17	0
CTC_ACT_58	315	1 (N=75)	30	0.0037	45	0.0730
		0 (N=17)	0		17	
CAC_ACA_071	215	1 (N=17)	0	0.0037	17	0.1005
		0 (N=76)	30		46	
CAC_ACA_075	222	1 (N=17)	0	0.0037	17	0.1005
		0 (N=76)	30		46	
CTG_AGG_63	249	1 (N=17)	0	0.0037	17	0.1005
		0 (N=76)	30		46	
CAG_AAC_109	319	1 (N=17)	0	0.0037	17	0.1005
		0 (N=76)	30		46	
CAT_AAC_73	283	1 (N=16)	0	0.0038	16	0.0346
		0 (N=67)	30		37	
CAA_ACC_19	104	1 (N=23)	1	0.0046	22	0.0782
		0 (N=70)	29		41	
CAA_AGG_104	333	1 (N=22)	1	0.0047	21	0.0758
		0 (N=71)	29		42	
CAC_ACA_028	122	1 (N=54)	25	0.0048	29	0.0561
		0 (N=39)	5		34	
CAA_ACA_54	282	1 (N=38)	5	0.0052	33	0.0702
		0 (N=54)	25		29	
CTG_AAC_069	197	1 (N=18)	0	0.0054	18	0.1152
		0 (N= 71)	26		45	
CTC_ACA_072	214	1 (N=17)	0	0.0056	17	0.1054
		0 (N=72)	27		45	

CAG_AAC_055	162	1 (N=52)	0	2.13E-11	52	5.01E-06
		0 (N=41)	30		11	
CTC_ACA_014	98	1 (N=28)	25	4.75E-11	3	3.66E-07
		0 (N=61)	2		59	
CAG_AGG_08	95	1 (N=47)	0	4.82E-10	47	1.03E-04
		0 (N=45)	30		15	
CAG_ACC_40	228	1 (N=46)	0	6.69E-10	46	9.35E-05
		0 (N=45)	30		15	
CAC_AAG_049	172	1 (N=22)	22	6.76E-09	0	4.81E-08
		0 (N=69)	7		62	
CAG_ACA_21	110	1 (N=27)	24	1.36E-08	3	2.03E-06
		0 (N=65)	6		59	
CTA_ACG_52	237	1 (N=37)	0	4.16E-08	37	1.73E-03
		0 (N=46)	30		16	
CTA_AGG_44	220	1 (N=72)	9	4.34E-08	63	1.63E-07
		0 (N=21)	21		0	
CAA_ACC_41	172	1 (N=47)	29	6.03E-08	18	5.62E-04
		0 (N=46)	1		45	
CAT_ACC_24	121	1 (N=48)	29	8.18E-08	19	8.92E-04
		0 (N=45)	1		44	
CAG_ACG_04	84	1 (N=40)	0	8.86E-08	40	1.39E-03
		0 (N=52)	29		23	
CTT_ACA_58	268	1 (N=71)	8	1.58E-07	63	2.71E-06
		0 (N=17)	17		0	
CTT_AAC_3	143	1 (N=54)	30	1.84E-	24	2.41E-

CAC_ACT_50	284	1 (N=80)	18	0.0002	62	0.0004
		0 (N=12)	12		0	
CAG_ACG_20	148	1 (N=31)	1	0.0002	30	0.0157
		0 (N=61)	28		33	
CTC_AAG_77	289	1 (N=64)	11	0.0002	53	0.0054
		0 (N=28)	19		9	
CAG_ACG_05	87	1 (N=47)	5	0.0003	42	0.0161
		0 (N=45)	24		21	
CTA_ACG_58	278	1 (N=34)	3	0.0003	31	0.0115
		0 (N=49)	27		22	
CAG_AGG_59	272	1 (N=42)	4	0.0003	38	0.0151
		0 (N=50)	26		24	
CAC_ACC_25	152	1 (N=42)	4	0.0003	38	0.0163
		0 (N=51)	26		25	
CAC_ACA_055	179	1 (N=46)	5	0.0003	41	0.0161
		0 (N=47)	25		22	
CAT_AAC_20	112	1 (N=64)	30	0.0004	34	0.0360
		0 (N=20)	0		20	
CAC_ACT_48	274	1 (N=81)	19	0.0004	62	0.0006
		0 (N=11)	11		0	
CAT_ACA_100	298	1 (N=65)	29	0.0005	36	0.0382
		0 (N=28)	1		27	
CTT_ACC_24	135	1 (N=27)	1	0.0005	26	0.0346
		0 (N=64)	29		35	
CTG_AAC_0	240	1 (N=24)	0	0.0005	24	0.063

CTC_ACT_07	92	1 (N=77)	19	0.0057	58	0.0244
		0 (N=15)	11		4	
CAG_ACA_26	124	1 (N=76)	30	0.0062	46	0.0924
		0 (N=16)	0		16	
CAG_ACA_35	143	1 (N=76)	30	0.0062	46	0.0924
		0 (N=16)	0		16	
CAG_ACC_17	135	1 (N=59)	12	0.0066	47	0.0309
		0 (N=32)	18		14	
CAG_ACC_37	214	1 (N=33)	4	0.0075	29	0.0825
		0 (N=58)	26		32	
CTA_AAC_26	123	1 (N=71)	29	0.0076	42	0.0944
		0 (N=21)	1		20	
CTT_ACC_30	148	1 (N=37)	5	0.0082	32	0.0680
		0 (N=54)	25		29	
CTC_ACA_076	227	1 (N=35)	4	0.0093	31	0.0917
		0 (N=54)	23		31	
CAT_ACA_001	78	1 (N=78)	30	0.0103	48	0.1203
		0 (N=15)	0		15	
CAC_AAC_46	230	1 (N=52)	23	0.0114	29	0.0941
		0 (N=38)	5		33	
CAC_AGG_55	220	1 (N=33)	4	0.0118	29	0.0873
		0 (N=60)	26		34	
CAC_ACA_064	201	1 (N=24)	2	0.0121	22	0.1124
		0 (N=69)	28		41	
CTT_AAC_6	194	1 (N=55)	11	0.013	44	0.067

6		0 (N=37)	0	07	37	03
CTG_AAC_070	202	1 (N=58)	4	2.49E-07	54	4.71E-04
		0 (N=31)	22		9	
CTG_AAC_003	77	1 (N=17)	17	3.15E-07	0	2.67E-06
		0 (N=72)	9		63	
CAG_ACA_22	111	1 (N=21)	20	3.85E-07	1	2.80E-06
		0 (N=71)	10		61	
CAG_AGG_119	121	1 (N=55)	30	3.99E-07	25	2.60E-03
		0 (N=37)	0		37	
CAA_AAC_18	117	1 (N=37)	0	4.02E-07	37	2.82E-03
		0 (N=56)	30		26	
CTT_ACA_40	177	1 (N=72)	9	5.41E-07	63	1.42E-06
		0 (N=16)	16		0	
CTT_ACA_41	179	1 (N=72)	9	5.41E-07	63	1.42E-06
		0 (N=16)	16		0	
CTC_AAG_38	178	1 (N=70)	10	8.55E-07	60	1.66E-05
		0 (N=22)	20		2	
CTC_ACG_2	193	1 (N=53)	27	9.71E-	26	6.67E-

86		0 (N=65)	26		39	5
CTT_AAC_76	224	1 (N=51)	26	0.0006	25	0.0200
		0 (N=40)	4		36	
CAG_AAC_028	115	1 (N=48)	6	0.0007	42	0.0225
		0 (N=45)	24		21	
CTA_ACG_51	230	1 (N=39)	5	0.0007	34	0.0132
		0 (N=44)	25		19	
CTA_ACG_49	213	1 (N=27)	1	0.0009	26	0.0253
		0 (N=66)	29		37	
CAC_ACT_34	167	1 (N=82)	20	0.0009	62	0.0016
		0 (N=10)	10		0	
CAC_ACT_52	330	1 (N=82)	20	0.0009	62	0.0016
		0 (N=10)	10		0	
CTC_ACA_077	231	1 (N=68)	27	0.0010	41	0.0712
		0 (N=21)	0		21	
CTG_AAC_031	118	1 (N=61)	25	0.0011	36	0.0575
		0 (N=28)	1		27	
CTG_ACC_4	172	1 (N=20)	0	0.0012	20	0.065

2		0 (N=36)	19	7	17	0
CAC_AAG_054	179	1 (N=40)	6	0.0138	34	0.0961
		0 (N=51)	23		28	
CTC_ACA_088	269	1 (N=14)	0	0.0157	14	0.1601
		0 (N=75)	27		48	
CTC_ACA_089	274	1 (N=74)	27	0.0165	47	0.1270
		0 (N=15)	0		15	
CAG_ACA_48	167	1 (N=13)	0	0.0167	13	0.0989
		0 (N=79)	30		49	
CAG_ACA_38	149	1 (N=79)	30	0.0167	49	0.1415
		0 (N=13)	0		13	
CAG_ACA_44	160	1 (N=79)	30	0.0167	49	0.1415
		0 (N=13)	0		13	
CTC_AAG_67	261	1 (N=79)	30	0.0167	49	0.1415
		0 (N=)13	0		13	
CTA_ACG_35	177	1 (N=80)	30	0.0168	50	0.1434
		0 (N=13)	0		13	
CAT_ACA_0	128	1 (N=63)	14	0.018	49	0.105

9				07		03
		0 (N=37)	0		37	
CTG_ACA_87	275	1 (N=35)	0	1.08E-06	35	4.07E-03
		0 (N=58)	30		28	
CAG_AAC_027	112	1 (N=58)	30	1.08E-06	28	4.07E-03
		0 (N=35)	0		35	
CTG_ACA_52	176	1 (N=45)	2	1.18E-06	43	2.22E-03
		0 (N=48)	28		20	
CTT_ACA_28	141	1 (N=71)	9	1.27E-06	62	3.78E-05
		0 (N=17)	16		1	
CTT_AAC_77	232	1 (N=33)	0	2.58E-06	33	4.89E-03
		0 (N=58)	30		28	
CAG_ACA_13	97	1 (N=75)	13	3.42E-06	62	4.49E-06
		0 (N=17)	17		0	
CTT_ACA_13	103	1 (N=72)	10	3.99E-06	62	5.72E-05
		0 (N=16)	15		1	
CAA_AAC_13	102	1 (N=62)	30	6.69E-06	32	1.05E-02
		0 (N=31)	0		31	
CTT_ACA_3	168	1 (N=49)	3	7.84E-	46	5.20E-

6						6
		0 (N=72)	29		43	
CTG_ACC_47	174	1 (N=21)	0	0.0012	21	0.0515
		0 (N=71)	29		42	
CTA_ACG_22	134	1 (N=20)	0	0.0012	20	0.0639
		0 (N=73)	30		43	
CAG_AAC_103	284	1 (N=20)	0	0.0012	20	0.0639
		0 (N=73)	30		43	
CAG_AAC_104	285	1 (N=20)	0	0.0012	20	0.0639
		0 (N=73)	30		43	
CAG_ACC_59	299	1 (N=20)	0	0.0013	20	0.0616
		0 (N=71)	30		41	
CAG_AAC_018	101	1 (N=76)	30	0.0013	46	0.1005
		0 (N=17)	0		17	
CTT_ACA_27	139	1 (N=68)	12	0.0013	56	0.0243
		0 (N=20)	13		7	
CTA_AGG_34	198	1 (N=63)	28	0.0014	35	0.0433
		0 (N=30)	2		28	
CAG_ACC_4	239	1 (N=74)	17	0.0016	57	0.012

26				2		2
		0 (N=30)	16		14	
CAC_AGG_15	109	1 (N=32)	4	0.0192	28	0.1108
		0 (N=61)	26		35	
CAT_ACC_08	89	1 (N=74)	29	0.0204	45	0.1178
		0 (N=19)	1		18	
CTA_ACC_49	190	1 (N=81)	22	0.0216	59	0.1134
		0 (N=11)	8		3	
CTC_AGG_73	314	1 (N=28)	3	0.0227	25	0.1338
		0 (N=63)	25		38	
CAG_ACG_24	193	1 (N=76)	19	0.0244	57	0.1323
		0 (N=16)	10		6	
CTG_ACT_39	175	1 (N=81)	30	0.0269	51	0.1842
		0 (N=12	0		12	
CTC_ACT_25	132	1 (N=80)	30	0.0270	50	0.1341
		0 (N=12)	0		12	
CAA_ACG_68	254	1 (N=72)	30	0.0271	42	0.1070
		0 (N=11)	0		11	
CTC_ACA_0	82	1 (N=42)	7	0.032	35	0.162

6		0 (N=39)	22	06	17	03
CTG_AAC_080	227	1 (N=50)	25	9.48E-06	25	1.06E-02
		0 (N=39)	1		38	
CAG_ACT_46	259	1 (N=30)	0	1.32E-05	30	1.04E-02
		0 (N=63)	30		33	
CAG_AAC_073	202	1 (N=30)	0	1.32E-05	30	1.04E-02
		0 (N=63)	30		33	
CTC_ACC_36	189	1 (N=30)	0	1.32E-05	30	1.04E-02
		0 (N=63)	30		33	
CAG_ACT_23	156	1 (N=54)	6	1.39E-05	48	9.59E-03
		0 (N=39)	24		15	
CTT_AAG_108	331	1 (N=19)	17	1.64E-05	2	1.26E-04
		0 (N=73)	13		60	
CAG_ACA_31	132	1 (N=24)	19	1.98E-05	5	6.98E-04
		0 (N=68)	11		57	
CTA_AGG_27	172	1 (N=29)	0	2.77E-05	29	0.0091
		0 (N=64)	30		34	
CTA_ACG_63	336	1 (N=26)	0	2.81E-05	26	0.0073
		0 (N=57)	30		27	
CTG_AGC_36	171	1 (N=65)	30	2.93E-05	35	0.0187
		0 (N=28)	0		28	
CTT_ACA_37	169	1 (N=69)	10	3.56E-05	59	0.0019
		0 (N=19)	15		4	
CTC_AAG_88	391	1 (N=69)	12	5.01E-05	57	0.0011
		0 (N=23)	18		5	

4		0 (N=17)	13		4	7
CTT_ACA_23	125	1 (N=21)	0	0.0016	21	0.1022
		0 (N=67)	25		42	
CTG_ACA_76	233	1 (N=80)	19	0.0017	61	0.0097
		0 (N=13)	11		2	
CAG_AAC_054	161	1 (N=11)	10	0.0017	1	0.0057
		0 (N=82)	20		62	
CAC_AAG_002	77	1 (N=72)	29	0.0020	43	0.0619
		0 (N=19)	0		19	
CAC_AAG_063	195	1 (N=72)	29	0.0020	43	0.0619
		0 (N=19)	0		19	
CTG_AAC_043	139	1 (N=61)	10	0.0022	51	0.0408
		0 (N=28)	16		12	
CAG_AAC_032	121	1 (N=75)	30	0.0022	45	0.0777
		0 (N=18)	0		18	
CAC_ACC_23	142	1 (N=75)	30	0.0022	45	0.0777
		0 (N=18)	0		18	
CAA_AAC_50	241	1 (N=75)	30	0.0022	45	0.0777
		0 (N=18)	0		18	
CTC_ACT_11	101	1 (N=19)	0	0.0022	19	0.0594
		0 (N=73)	30		43	
CTC_ACT_42	206	1 (N=19)	0	0.0022	19	0.0594
		0 (N=73)	30		43	
CTC_ACT_46	216	1 (N=19)	0	0.0022	19	0.0594
		0 (N=73)	30		43	

04		0 (N=47)	20	8	27	0
CTC_ACT_05	88	1 (N=78)	21	0.0373	57	0.1547
		0 (N=14)	9		5	
CTT_ACA_53	231	1 (N=75)	25	0.0418	50	0.2111
		0 (N=13)	0		13	
CTG_AAC_057	175	1 (N=67)	24	0.0419	43	0.1914
		0 (N=22)	2		20	
CTG_ACA_93	322	1 (N=82)	30	0.0433	52	0.1704
		0 (N=11)	0		11	
CAG_AAC_108	299	1 (N=82)	30	0.0433	52	0.1704
		0 (N=11)	0		11	
CTG_AGG_36	179	1 (N=11)	0	0.0433	11	0.1704
		0 (N=82)	30		52	
CAG_ACA_46	163	1 (N=11)	0	0.0435	11	0.1683
		0 (N=81)	30		51	
CTT_ACC_13	102	1 (N=80)	30	0.0439	50	0.1662
		0 (N=11)	0		11	
CTT_AAC_25	121	1 (N=80)	30	0.0439	50	0.1662
		0 (N=11)	0		11	
CAT_ACC_98	345	1 (N=70)	29	0.0505	41	0.1542
		0 (N=15)	1		14	
CAG_ACA_58	182	1 (N=70)	18	0.0518	52	0.1802
		0 (N=22)	12		10	
CTG_AAC_062	183	1 (N=27)	3	0.0518	24	0.2166
		0 (N=62)	23		39	

CAT_ACC_97	344	1 (N=46)	27	5.54E-05	19	0.0042	CTT_AAG_074	196	1 (N=41)	22	0.0026	19	0.0296	CAT_ACA_048	168	1 (N=62)	25	0.0542	37	0.1835
		0 (N=39)	3		36				0 (N=51)	8		43				0 (N=31)	5		26	

The statistical significance was evaluated using Fisher´s exact probability test. Fragments are listed from the most significant to the least significant ones for healthy donors.

Identification of AFLP markers using NGS

To evaluate the data obtained by NGS, two approaches in NextGENe software v2.4.1 (Softgenetics, State College, PA, USA) were applied, the amplicon and shotgun sequencing. The number of reads provided by both approaches are listed in Table C.

Table C. Number of reads using the amplicon and shotgun approach to data evaluation after NGS.

AFLP marker	MID	Number of reads	
		Amplicon sequencing	Shotgun sequencing
CAG_AGG_56	1	1610	2525
CAT_ACC_54	2	1283	2077
CAG_ACC_32	3	2577	3645
CAC_AAG_063	4	2646	3909
CTT_ACA_57	6	7772	10019
CAA_ACC_41<br>CAA_ACC_19	7	2257	3394
CAC_ACC_25	8	4116	5803
CAA_AAC_50	10	4485	6483
CTT_ACA_57	11	1690	3149

MID - molecular identifier tag

Validation of sequences and identified polymorphisms by *in silico* analyses and Sanger sequencing

To perform virtual DNA cutting and fingerprinting in *in silico* fingerprinting (ISIF) software, we used parameters as shown in Table D. The sequences obtained by both approaches, *in silico* AFLP and NGS, were confirmed by Sanger sequencing with designed primers, and their characteristics are shown in Table E.

Table D. Parameters for *in silico* fingerprinting using ISIF software.

AFLP marker	Fragment length (bp)	Length range (bp)	Selective bases (RC)	Left cut	Right cut	Selective bases	5´Primer length (bp)	3´Primer length (bp)
CAG_ACC_32	188	183-193	GGT	G	AATTC	ACC	11	15
			CTG	T	TAA	CAG	13	15
CTT_ACA_57	257	252-262	TGT	G	AATTC	ACA	11	15
			AAG	T	TAA	CTT	13	15
CAG_AGG_56	263	258-268	CCT	G	AATTC	AGG	11	15
			CTG	T	TAA	CAG	13	15
CAA_ACC_41	172	167-177	GGT	G	AATTC	ACC	11	15
			TTG	T	TAA	CAA	13	15
CAC_ACC_25	152	147-157	GGT	G	AATTC	ACC	11	15
			GTG	T	TAA	CAC	13	15
CAC_AAG_063	195	190-200	CTT	G	AATTC	AAG	11	15
			GTG	T	TAA	CAC	13	15
CAA_AAC_50	242	237-249	GTT	G	AATTC	AAC	11	15
			TTG	T	TAA	CAA	13	15
CAA_ACC_19	104	99-109	GGT	G	AATTC	ACC	11	15
			TTG	T	TAA	CAA	13	15
CAT_ACC_54	175	170-180	GGT	G	AATTC	ACC	11	15
			ATG	T	TAA	CAT	13	15

The parameters needed for virtual fingerprinting were as follows: (a) the range of fragment length in basepairs (bp), (b) trinucleotide parts of amplification primers called selective bases (CXX for MseI and dye-labeled AXX for EcoRI; X stands for A, C, G or T) and their reverse complement sequences (RC); (c) “Left Cut” column corresponds to the part of the sequence in 5´ of the enzyme restriction

site, and the “Right Cut” column corresponds to the 3´ part of the sequence after the cleavage site, G|AATTC for EcoRI and T|TAA for MseI. (d) The length of both primers was calculated according the formula stated in [8].

Table E. Characteristics of primers used for Sanger sequencing of AFLP markers.

AFLP marker	Sequence	Chromosome	Forward primer	Reverse primer	Annealing temperature (°C)	Amplicon length (bp)
CAG_ACC_32	AATTCACCAGGCTGGAATTTCCTAATCCTAGCAAGCCTGTGGACACTGCCAGAGACTAGGGCATGTTTCATCCCTATCTACAACTGCATAAGGCAGACACTCCCAGAGTGGCCGTTTTAGAGGCTCCCCTCCCCCGAGGAGTGTATTCTTTTCCCAGGGCTGT	Y	AGACTCTGTATGACCCATAGTT	ACGAAATCTTGGCACCTTGA	62	940
CTT_ACA_57	TAACTTGAGATACAAATTAGACAGAAGCATTCTCAGGAACTGCTTTGTGATGTGTGCATTCAACTCACGGACTTGAACCTTCCCTTTGAGAGAGTCGGTTTTAGAAACAGTTCTTTTGTAGTATCTGAAATTGGATATTTAGAGCGACTTGAGTCCTATGATAGGAAAGGAATACTCCTCACATAAAAATTGGACAGAAGCATTCTCAGAAACTTCTTCGTGATATGTG	5	n.a.	n.a.	n.a.	n.a.
CAG_AGG_56	AATTCAGGCCAACCATCAAAAGAGACCAAAAAATGGCACTATATTGCTAAGGGTACGATTCACAATGAAAACAAAACAGCTATAAATGTTTGATGCCCATATTTAGAGTTTATTGATACTTTTGTAAGAAGAGAGATCATACTTCATAGGGAACTACAGAATGTCTCAGTAAGTTAGGCAGGGCTTGTTATAGGATTGGGGTCTGTTATAGGGTTCAAGGACCTGTGGTTTTGCTGT	14	CACAAAATGACAGAACTAAGACCA	CCCTGTGACAACAATGGAGT	63.5	723
	AATTCAGGGCAAGGGTGGGGAGGGCTTGGCCTTACAGGTTCTTGGCTTAGAGGAGGTGAGACTGGGGCTGATGGAGGAGCAGGATAACTCTTCTCTAGATCTCTTTCCTGTAGATGGTTTGTATGTACAAATAAGGCAGATTGTGAGATTTCTGTATTGGGGTAGAGTCATTCAGGCTGTTGAGATGAGCTGCATTTACTTTAGAGAGAATC	9	GAACTTTGACAGCATCCTCT	ATCCTCCTGCCTCACCTCAA	65	848

	ACATTTGCAGCATTACCTGT					
	AATTCAGGCCAACCATCAAGAGACCAAAAAATGGCACTATATTGCTAAGGGTA CGATTCACAATGAAAACAAAACAGCTATAAATGTTTGATGCCCATATTTAGAGT TTATTGATACTTTTGTAAGAAGAGAGATCATACTTCATAGGGAACTACAGAATG TCTCAGTAAGTTAGGCAGGGCTTGTTATAGGATTGGGGTCTGTTATAGGGTTC AAGGACCTGTGGTTTTGCTGT	16	GGCTTACAAAGGGCATCATTA	CTGACTTCAATACCATGTTG	55	640
CAA_ACC_41	AATTCACCAAACACACATCCTCTTTAAGGGCCAGTCAGGCTCTTTGACTGGTAA TACTTGACCTCATTTGTACCTTGTCTTCAAACTGCAGCAGAGTTCAGATCCCTGA TGCACAAAGCAATGCTCTTTTCACTTCTCCTTGTTGT	18	TTATCCACCACCACCATCTC	GAGAAAAAGCACGATGCCTA	63.5	587
	TAACAAGTAACGTCAATGCTCCAGTAGTGTGAAGAACTTCCATAAAGTACATTA GGGAAACAAGACAGGCATGGAGTAGATGTCATTTTGAAAACAGACTTCCCACT TTAGGTGGACCCTAAAGAATGGGAGTGGGGAGAAAGGTG	15	CTCTAGCGACCTGGGATTAC	ACTAAAGGCAAGCACAACTG	63.5	817
CAC_ACC_25	TAACACCAAGAGTGAGCCCTGGCAGGACATTATGGCTCATAATTGTAATCCCA GCACCATGGGAGACTGAGGCGGGCAGATCACTTGAGGTCAGGAGTTTGAGAC CAGCCTGGCCAACATGGTG	1	GAAGGGAGCTAATAAGCCATGAA	GTGTTCACAGTAAAATCGAGG	62	815
CAC_AAG_063	TAACACAAGGCAACAAGTCACAGGTGAAAATTGAGCCTCTGAGGCCCTGCTGA TTGCACAGGGGGGGGAAGGCATTTGCATCTCTTCTCACCACAGAGGGGTGGCAG CCAATTACCTCCAGGATTAGAAAAAAGTTGCAAACCTGAGTCTATCTTCACTGA CGGGCTTG	19	CAAGACTTACTTTCTCAGTC	GCAACATTTCTGCTTCTGGT	56.7	483

	TAACACAGTACCTGGTACAGAGTACATGCTCAATAAATTATGTGTCTGTCCATC TATCTATCGATCTATCTATTTATCTACTATCCATCCTACTTATATAAATCCCACTC CTCAACCCATCCTAACAGGCAGATGGACCATCTGCAGTCCCCCTGCTTCCACTT G	1	CAGCAAACCCTGACACTTCA	TCTCACTGCCACAATACAGC	55	603
CAA_AAC_50	AATTCAACAAGAGGATGCCTTCTCCTTTTGTCATGCTCAATGATACATTTAGGGT GCCTATTACATGTAGTGAAAATACTCATACAAGTTTTAGGAAAAATCCCCAGAC TCAGTTACACAAACATAAACTACCTTACTCATCAAAATAAAACAAAACAAAAAC AACAACAGTGAAAACTCTGAAAAGCATCATTCTGCCAAGATAATCACTTGTTG	5	GGAATGTACTGTGGCTTGTT	AGTTGATTTCACCCATCTGA	56.7	413
CAA_ACC_19	AATTCACCAATGAAGCCATCAGGTCTAAGGCTTTTCTTTGTCAGGAAGATTTTA TTATTGATTCAGTCTCTTTACTTGT	22	TAGGTATCAGGGCAATGAGT	AAGAGAAGCACAGAGTGGAG	63.5	648
CAT_ACC_55	AATTCACCAGCAACCAGAGGGGAGGGCTGGAGGGAAGATTCGGAGGGGCTG GCCCAAGAGCACCTATGAGCCCCTTTGGAAACCATCCCAATAGGGGAAGCTGG GGGACTTTTCTGGAATAAGGGGGAAGGTTCATGCTATAATCATGT	10	GGCTACCAGGTACATTCCAA	GTGGGTTGCCCTCATACTTA	62	917

n.a. – it was not possible to design primers

Difficulty in scoring of absence/presence of the AFLP markers

The inconsistence of data obtained through Sanger sequencing and fragment analysis was probably caused by the difficulties in scoring two overlapping fragments CAT_ACC_54 and CAT_ACC_55, and hence resulted in misrepresentation of the statistics after fragment analysis (Figure G and Table 2).

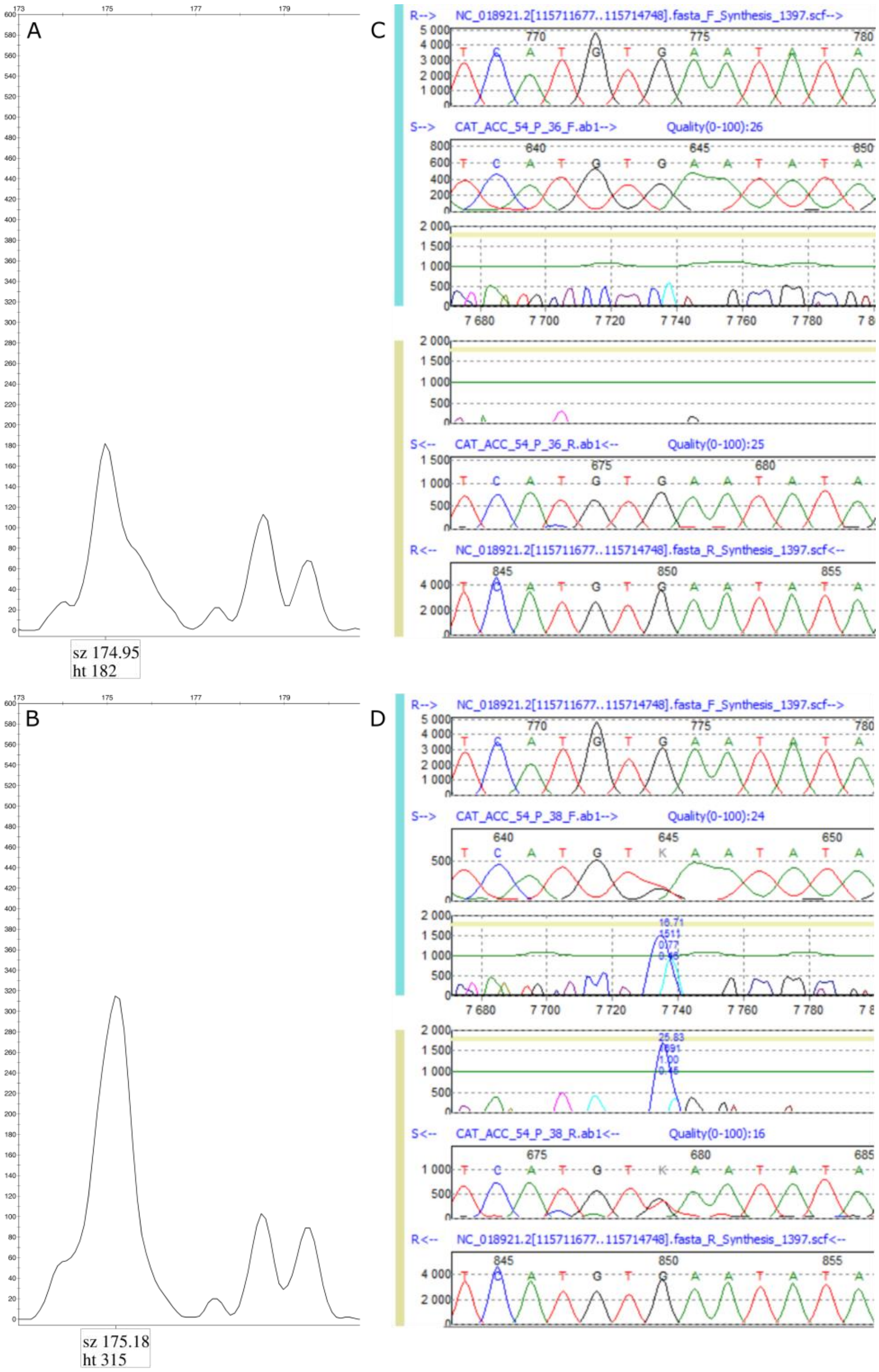

Figure G. The problematic scoring of two overlapping fragments CAT_ACC_54 and CAT_ACC_55. The presence of one-bp shorter fragment CAT_ACC_54 impeded the scoring and misrepresented statistics after the fragment analysis. Panel A and B show the presence of AFLP fragments CAT_ACC_54 and CAT_ACC_55, respectively, and the corresponded sequences analyzed by Sanger sequencing (Panel C and D). (D) The fragment CAT_ACC_55 was formed as a result of heterozygous substitution G/T (SNP rs7906704) leading to an introduction of a new MseI restriction site.

References

1. Valent P. Imatinib-resistant chronic myeloid leukemia (CML): Current concepts on pathogenesis and new emerging pharmacologic approaches. Biologics : targets & therapy. 2007;1: 433.

2. Cortes JE, Talpaz M, Giles F, O'Brien S, Rios MB, Shan J, et al. Prognostic significance of cytogenetic clonal evolution in patients with chronic myelogenous leukemia on imatinib mesylate therapy. Blood. 2003;101: 3794-3800. doi: 10.1182/blood-2002-09-2790.

3. Gorre ME, Mohammed M, Ellwood K, Hsu N, Paquette R, Rao PN, et al. Clinical resistance to STI-571 cancer therapy caused by BCR-ABL gene mutation or amplification. Science. 2001;293: 876-880. doi: 10.1126/science.1062538.

4. Donato NJ, Wu JY, Stapley J, Lin H, Arlinghaus R, Aggarwal B, et al. Imatinib Mesylate Resistance Through BCR-ABL Independence in Chronic Myelogenous Leukemia. Cancer Research. 2004;64: 672-677. doi: 10.1158/0008-5472.CAN-03-1484.

5. Baccarani M, Deininger MW, Rosti G, Hochhaus A, Soverini S, Apperley JF, et al. European LeukemiaNet recommendations for the management of chronic myeloid leukemia: 2013. Blood. 2013;122: 872.

6. Vos P, Hogers R, Bleeker M, Reijans M, van de Lee T, Hornes M, et al. AFLP: a new technique for DNA fingerprinting. Nucleic acids research. 1995;23: 4407-4414. doi: 10.1093/nar/23.21.4407.

7. Prochazka M, Walder K, Xia J. AFLP fingerprinting of the human genome. Hum Genet. 2001;108: 59-65.

8. Paris M, Després L. In silico fingerprinting (ISIF): a user-friendly in silico AFLP program. Methods in molecular biology (Clifton, N.J.). 2012;888: 55.

9. Altshuler DM, Albers CA, Abecasis GR, et al. A global reference for human genetic variation. Nature. 2015;526: 68-74. doi: 10.1038/nature15393.

10. Lek M, Karczewski KJ, Minikel EV, Samocha KE, Posthuma D, Exome Aggregation Consortium [. Analysis of protein-coding genetic variation in 60,706 humans. Nature. 2016;536: 285-291. doi: 10.1038/nature19057.
